# Phytochemical Characterizations of *Maranthes polyandra* (Benth.) Prance

**DOI:** 10.3390/molecules27041316

**Published:** 2022-02-15

**Authors:** Nida Ali, Farooq-Ahmad Khan, Kayode Muritala Salawu, Rimsha Irshad, Almas Jabeen, Chun-Lei Zhang, Muhammad Iqbal Choudhary, Xin-Min Liu, Yan Wang

**Affiliations:** 1 H. E. J. Research Institute of Chemistry, International Center for Chemical and Biological Sciences, University of Karachi, Karachi 75270, Pakistan; s.nidaali.m@gmail.com (N.A.); farooq.khan@iccs.edu (F.-A.K.); rimshairshad62@gmail.com (R.I.); iqbal.choudhary@iccs.edu (M.I.C.); 2 Sino-Pakistan Cooperation Center for Traditional Chinese Medicine, Hunan University of Medicine, Huaihua 418000, China; 3 Sino-Pakistan Cooperation Center for Traditional Chinese Medicine, International Center for Chemical and Biological Sciences, University of Karachi, Karachi 75270, Pakistan; 4 Third World Center (TWC) for Chemical Sciences, International Center for Chemical & Biological Sciences, University of Karachi, Karachi 75270, Pakistan; 5 Department of Pharmacognosy and Drug Development, University of Ilorin, Ilorin 240003, Nigeria; pharmmks@yahoo.com; 6 Dr. Panjwani Center for Molecular Medicine and Drug Research, International Center for Chemical and Biological Sciences, University of Karachi, Karachi 75270, Pakistan; almas79_jabeen@yahoo.com; 7 School of Traditional Chinese Pharmacy, China Pharmaceutical University, Nanjing 211198, China; zhangchunlei11@sina.com; 8 Institute of Medicinal Plant Development, Chinese Academy of Medical Sciences, Beijing 100193, China

**Keywords:** Chrysobalanaceae, *Maranthes polyandra*, *Parinari polyandra*, triterpenoid, GC-MS

## Abstract

Two new ursane-type triterpenoids, named Polyanside A (**1**) and B (**2**), along with eleven known compounds (**3**–**13**), were isolated and elucidated from *Maranthes polyandra* (Benth.) Prance. The structures of these compounds were elucidated based on chemical evidence and multiple spectroscopic data. Isolated compounds were evaluated for anti-cancer, anti-inflammatory activities, and cytotoxicity on a normal human cell line (BJ). None of them showed activity and cytotoxicity. The hexane fraction was analyzed by GC-MS, resulting in the identification of forty-one compounds. This is the first comprehensive study on the phytochemistry of *M. polyandra*.

## 1. Introduction

The history of medicinal plants is as old as the history of human beings. Natural products have played a vital role in drug discovery. The use of natural components from folk medicines requires a clear understanding of chemistry, efficacy, and safety. Now, there has been a surge in interest in valorizing the biological importance of medicinal plants [1,2]. It is a pressing priority to obtain potent phytoconstituent from different medicinal plants and to explore their promising benefits [3,4].

*Maranthes polyandra* (Benth.) Prance (Synonym: *Parinari polyandra* Benth., World Flora Online) belongs to the Chrysobalanaceae family. It is a savannah tree of Africa ranging from Mali to Sudan, some parts of southern states of Nigeria, and largely found in Benin, where it is locally known as Wantuwiwi [5,6]. Different parts of this tree have been used for various ailments, for example, measles [7], diarrhea [8], fertility disorder [9,10], wounds, fracture, fever, and syphilis [11,12]. The extract from the stem bark, fruit, and seed of *M. polyandra* has shown anti-inflammatory and antinociceptive [9], antihyperlipidemic, hypercalcemic [10], hypertensive, anti-hypercholesterolemia, anti-diabetic [13], and antioxidant effects [14]. However, phytochemistry investigation of this species is extremely limited, except for a few studies [15,16,17,18]. Until now, only three compounds (xanthoxylin, β-eudesmol, luteolin) have been isolated from this plant [15]. The composition of seed oil has been analyzed by GC-MS [17,18]. In addition, GC-MS analysis on the extract and fractions of *M. polyandra* stem bark just confirmed the presence of some fatty acids [16].

Thus, the current study aimed to explore the phytochemical constituent from *M. polyandra* through isolation and GC-MS analysis. Finally, two new triterpenoids (**1** and **2**), and eleven known compounds (**3**–**13**) were isolated. In addition, GC-MS analysis of the hexane fraction also led to the identification of 41 compounds. This is the first comprehensive phytochemistry study of this species. Anti-cancer and anti-inflammatory activities and cytotoxicity of compounds **1**, **2**, **5**, **6**, **8**, **11,** and **13** were evaluated. None of them were active. Based on traditional uses, anti-inflammatory components might exist in this plant. Further study may be required to discover potent anti-inflammatory molecules from this species.

## 2. Results and Discussion

### 2.1. Structure Elucidation of Isolated Compounds

**Figure 1:**
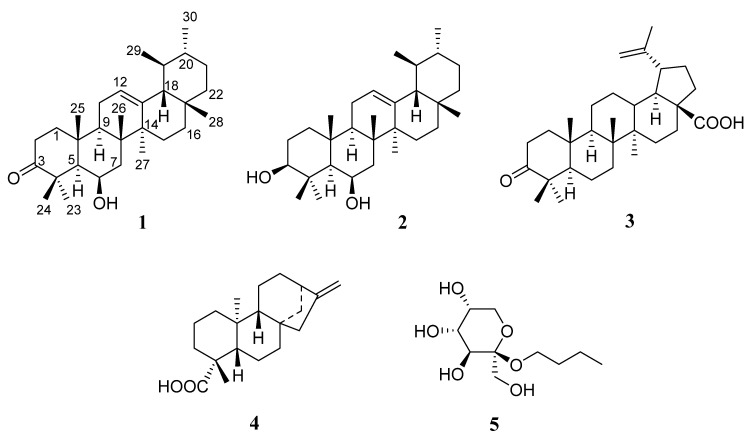
Structures of compounds **1**–**5**.

First, 80% MeOH extract of the stem bark of *M. polyandra* was fractioned by *n*-hexane for GC-MS analysis. The remaining residue was then isolated using chromatographic techniques, such as silica gel column chromatography (CC), C18 CC, Sephadex LH 20 CC, and HPLC. Thirteen compounds (**1**–**13**) were obtained, including two new compounds (**1** and **2**) and eleven known compounds (**3**–**13**) (Figure 1). The structures of **1** and **2** were elucidated mainly through NMR techniques, primarily based on 1D NMR (^1^H and ^13^C NMR), 2D NMR (COSY, HSQC, HMBC, and NOESY), and MS techniques including EI-MS and HR-EI-MS.

**Figure 2:**
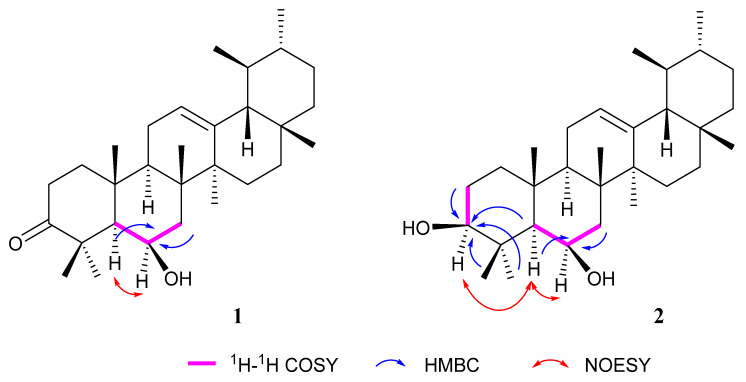
Key ^1^H-^1^H COSY, HMBC, and NOESY correlations of compound **1** and **2**.

Polyanside A (**1**) was obtained as needle-shaped white crystals. The molecular formula was recognized as C_30_H_48_O_2_ based on HR-EI-MS (*m*/*z* 440.3648 [M]^+^, calcd. for 440.3654), representing seven degrees of unsaturation (Figure S2). 1D NMR (Table 1, Figures S6–S13) revealed the presence of thirty carbons, including eight methyl groups at *δ*_H_ 1.50, 1.40, 1.35, 1.15, 1.02, and 0.80 as singlets, along with a broad singlet at *δ*_H_ 0.90 (br s) and a doublet at *δ*_H_ 0.78 (d, *J* = 5.8 Hz). A group of typical signals consisting of an olefinic proton at *δ*_H_ 5.18 (dd, *J* = 5.0, 2.5 Hz), two olefinic carbons at *δ*_C_ 124.5, and 139.0, and a carbonyl signal at *δ*_C_ 216.7, were suggestive of a urs-12-en-3-one skeleton. All NMR data showed great similarity with α-amyrone except an extra oxymethine signal at *δ*_H_ 4.49 (br s) that is correlated with *δ*_C_ 69.3 in HSQC [19,20]. The presence of a hydroxyl was confirmed at C-6 through COSY correlations between H-5 (*δ*_H_ 1.22), H-7a (*δ*_H_ 1.81, dd, 14.8, 3.8), H-7b (*δ*_H_ 1.55, overlapped), and *δ*_H_ 4.49, along with HMBC correlations between H-5 (*δ*_H_ 1.22), H-7a (*δ*_H_ 1.81), H-7b (*δ*_H_ 1.55), and *δ*_C_ 69.3. The orientation of the hydroxyl can be confirmed as β, because correlations between H_3_-25 (*δ*_H_ 1.50), H_3_-26 (*δ*_H_ 1.35) and H-6 (*δ*_H_ 4.49) were absent; instead, correlation between H-5 (*δ*_H_ 1.22) and H-6 (*δ*_H_ 4.49) was observed. Thus, the structure of compound **1** was elucidated as shown in Figure 1 and named Polyanside A. Key ^1^H-^1^H COSY, HMBC, and NOESY correlations are shown in Figure 2 (Figures S14–S21).

Polyanside B (**2**) was obtained as an amorphous white powder with molecular formula of C_30_H_50_O_2_ deduced by HREIMS (*m*/*z* 442.3832 [M]^+^, calcd. for 442.3811), representing six degrees of unsaturation (Figure S23). 1D NMR data (Table 1, Figures S27–S33) of **2** is in good agreement with **1**, except one more oxymethine proton at *δ*_H_ 3.14 (dd, *J* = 10.0, 5.0 Hz) and the absence of a carbonyl signal. The location of *δ*_H_ 3.14 was confirmed at C-3 through HMBC correlations of *δ*_H_ 3.14 with C-2 (*δ*_C_ 27.4), C-5 (*δ*_C_ 55.5), C-23 (*δ*_C_ 17.0), and C-24 (*δ*_C_ 28.05), along with COSY correlations between *δ*_H_ 3.14 and H-2 (*δ*_H_ 1.61 and 1.63). In addition, H-3 exhibited correlations with H-5 (*δ*_H_ 0.74, d, 2.0 Hz), implying a β-orientation of the hydroxyl at C-3. Therefore, the structure of compound **2** was elucidated as shown in Figure 1 and named Polyanside B. Key COSY, HMBC, and NOESY correlations are shown in Figure 2 (Figures S34–S41).

Compounds **3**–**13** were isolated from *M. polyandra* for the first time and recognized by compared to previously reported data. They were betulonic acid (**3**) [21], kaur-16-en-19-oic acid (**4**) [22], *n*-butyl-β-D-fructopyranoside (**5**) (Figure 1) [23], β-sitosterol (**6**) [24,25], stigmasterol (**7**) [26,27], stigmastane-3,6-dione (**8**) [28], stigmastane-4-ene-3-one (**9**), 4,22-stigmastadiene-3-one (**10**) [29], β-sitosterol β-D-glucoside (**11**) [30], *n*-hexadecanol (**12**), and palmitic acid (**13**) [31].

Compounds **1**, **2**, **5**, **6**, **8**, **11,** and **13** were performed for anti-cancer activity against MCF-7 cell (breast cancer), NCI-H460 (lung cancer), Hela (cervical cancer), and cytotoxicity against normal human cell line BJ, which were obtained from a cell culture biobank (PCMD, ICCBS) of American Type Culture Collection (ATCC), MTT assay was used for this activity (S3.5) [32]. All of them were observed to be inactive and nontoxic with inhibition < 50% at 50 µM. Compounds **1**, **2**, **5**, **6**, **8**, **11**, and **13** were also screened for nitric oxide (NO) inhibitory activity by a previously described method (S3.6) [33]. Unfortunately, all tested compounds displayed <50% inhibition at 25 µg/mL. The methanol extract and hexane fraction were tested for the same assays. However, they were inactive.

Compound **6** was reported to possess a good antinociceptive effect conferring to hot-plate and tail-flick assays [34]. Compounds **6** and **11** have been claimed to be the responsible components of an active extract to inhibit the growth of A549 cells (lung carcinoma epithelial cells) by analyzing the extract by LC-MS-MS [35]. However, in the current study they were inactive against NCI-H460 (lung cancer). Sari et al. evaluated the antimicrobial potential of **6** and **8**. It was observed that **6** inhibited *S. aureus* with MIC of 9.4 µg/mL. Meanwhile, **8** inhibited *S. enterica* with MIC of 37.5 µg/mL [36]. To the best of our knowledge, it is the first time to test compounds **1**, **2**, **5**, **6**, **8**, **11**, and **13** for their anti-cancer potential (against MCF-7, HeLa, and H460) as pure compounds.

### 2.2. Phytochemical Investigation of Hexane Fraction by GC-MS

**Figure 3:**
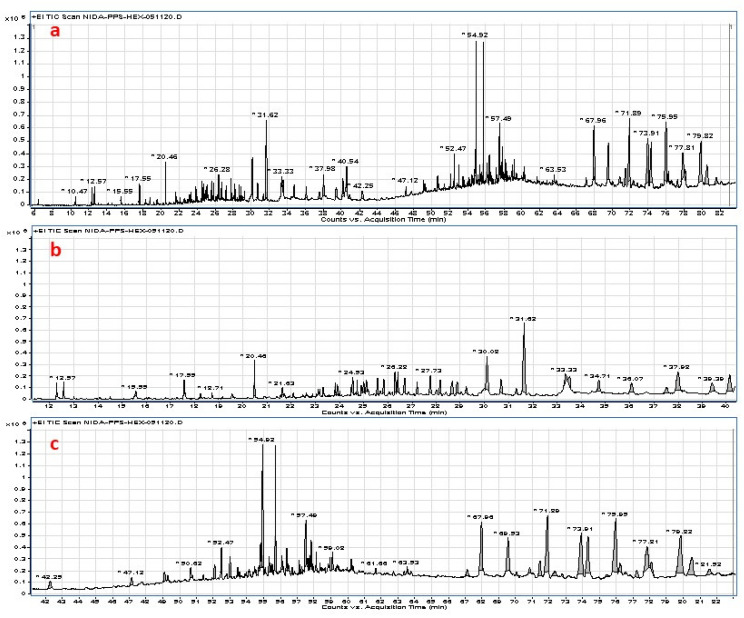
GC chromatogram of hexane fraction, (**a**): GC chromatogram of 5–84 min; (**b**): GC chromatogram of 11–40 min; (**c**): GC chromatogram of 41–84 min.

GC-MS analysis of the hexane fraction revealed the presence of different phytochemicals, which are shown in Figure 3 and listed in Table 2.

The major phytocomponents obtained from the hexane fraction were β-amyrin (8.55%), 4,22-stigmastadiene-3-one (8.33%), β-amyrin methyl ether (7.71%), stigmast-4-*en-*3-one (7.4%), stigmasterol (6.22%), *n-*hexadecanoic acid methyl ester (5.17%), (22*E*)-3 methoxystigmasta-5,22-diene (3.85%), 5*α*-stigmastane-3,6-dione (3.27%), γ-sitosterol (2.99%), methyl 13-octadecenoate (2.61%), methyl (10*E*)-10-octadecenoat (2.61%), oleyl alcohol (2.36%), trans-9-octadece*n-*1-ol (2.36%), hexadecanol (2.15%), 9-hexadece*n-*1-ol (2.15%), methyl linoleate (2.07%), 1-heptadecanol (1.24%), 3-hydroxy-4-methoxybenzaldehyde, acetate (1.16%), eicosane (0.93%), and friedelan-3-one (0.92%).

By deciphering the results obtained from the GC-MS analysis, it was observed that *M. polyandra* contained various phytochemicals that are known for their different medicinal and economical importance. These results were acquired firstly through gas chromatogram, in which area of the peaks indicated the relative concentration of the phytoconstituent present in hexane fraction, and their structures were identified through NIST online database for mass spectrometry. The obtained phytochemicals have been reported to possess different biological activities, including antimicrobial, antioxidant, anti-inflammatory, and anti-cancer effects. These results provided new knowledge about the non-polar components from *M. polyandra*.

## 3. Materials and Methods

### 3.1. General Experimental Procedures

Low-resolution mass spectra EI-MS were chronicled on a JEOL MS route JMS 600H instrument, and HR-EI-MS was analyzed on Thermo Finnigan MAT 95XP linked with X-Calibur. The ^1^H and ^13^C NMR spectra were recorded on a Bruker Avance NEO-500, 400 NMR spectrometer in CDCl_3_ at 500, 400, and 125 MHz, respectively. The UV was checked on the Evolution^TM^ 300 Spectrophotometer, and FT-IR spectra were recorded on a Bruker Vector 22 spectrophotometer. Optical rotations were determined on a JASCO 2000 Polarimeter. The purity of the compounds was verified on TLC (Silica gel, Merck F254, 0.25 mm thickness). Melting points were determined in glass capillary tubes using the Buchi melting point apparatus. For the TLC plate’s visualization, vanillin and ceric sulfate staining reagents were used. All experiments were performed at room temperature using solvents acquired commercially and used without further purification.

### 3.2. Collection of Plant Material

The stem bark of *Maranthes polyandra* (Benth.) Prance was collected by Mr. Kayode Muritala Salawu, a Senior Lecturer in the Department of Pharmacognosy and Drug Development, University of Ilorin, Kwara State, Nigeria, in August 2018 in the main campus of the University of Ilorin. The plant was identified and authenticated at the Herbarium Unit of the Department of Plant Biology, University of Ilorin, where the voucher specimen was deposited by the synonym *Parinari polyandra* Benth and voucher number (UILH/001/582/2021) was issued.

### 3.3. Extraction and Isolation

The sample was washed properly with distilled water, then air-dried and ground. The powder (1.2 kg) was extracted with 80% of methanol by using a Soxhlet extractor. The extract was concentrated to dryness in vacuum. The residue (104.3 g) was suspended in water, and extracted by hexane. The hexane layer (1.2 g) was used for GC-MS analysis. The remaining residue was extracted by BuOH and the main fraction (38.4 g) was obtained, which was subjected to silica gel (100–200 mesh) column chromatography (CC) and eluted with Hexane/DCM/MeOH (100:0:0–0:0:100). Finally, 20 major fractions (F_1_–F_20_) were obtained. F_5_ (358.7 mg) was separated using silica gel CC and eluted with Hexane/EtOAc (99:1 to 1:1) to afford 10 sub-fractions (F_5-1_–F_5-10_). Then F_5-5_ was subjected to normal phase preparative HPLC (98% hexane/2% EtOAc) and **12** (5.0 mg) was obtained. F_7_ (450.5 mg) was performed on silica gel CC and eluted with Hexane/EtOAc to afford 14 sub-fractions (F_7-1_–F_7-14_). F_7-3_ (29.2 mg) was subjected to HPLC (Hex/EtOAc 9:1) and gave **9** (3.5 mg). F_7-4_ (25.5 mg) was chromatographed using HPLC (Hex/EtOAc 9:1) to give **1** (9.4 mg) and **10** (3.5 mg). F_7-8_ (80.8 mg) and F_7-9_ (26.4 mg) was subjected to HPLC (Hex/EtOAc 8:2) respectively to yield **6** (10.3 mg), **4** (2.7 mg), **7** (2.8 mg), and **8** (8.3 mg), respectively. While F_7-12_ (53.0 mg) and F_7-13_ (25.2 mg) were followed by silica gel CC, then subjected to Sephadex LH-20, and acquired five sub-fractions, respectively. F_7-12-2_ (29.2 mg) and F_7-13-2_ (19.5 mg) were purified by HPLC (Hex/EtOAc 7:3) to give **2** (3.0 mg) and **3** (3.5 mg), respectively. F_10_ (677.8 mg) was chromatographed on silica gel CC and get 10 sub-fractions (F_10-1_–F_10-10_). F_10-1_ (74.8 mg) was purified via HPLC using hexane: ethyl acetate (7:3), compound **13** (7.0 mg) was obtained. **5** (18.2 mg) was crystalized from F_15_ (650.5 mg), and the remaining residue was purified using silica gel CC (EtOAc/MeOH, 99:1–100:0) and gave **11** (8.2 mg).

Polyanside A (**1**): needle-like crystals. [α]^27^_D_ −23 (c 0.001, MeOH); UV (MeOH) λ_max_ 213 nm (logε) (2.52) (Figure S3); m.p. 260–262 °C; IR (KBR) *v*_max_ 3734 broad (O-H), 3263 (=C-H), 2919 (C-H), 1691 (C=O), 1453(C=C), 1058 (C-O), and 914 cm^−1^ (=C-H) (Figure S4); CD nm [mdeg] 370 (2.66), 356 (−0.37), 336 (0.80); 314 (−3.46), 206 (50.14) (Figure S5); ^1^H NMR (CDCl_3_ 500 MHz) and ^13^C NMR (CDCl_3_ 150 MHz) data, see Table 1; EI-MS *m*/*z* 440.4 [M]^+^ (Figure S1); HR-EI-MS (*m*/*z* 440.3648 [M]^+^ calcd. for C_30_H_48_O_2_ 440.3654) (Figure S2).

Polyanside B (**2**): an amorphous powder. [α]^27^_D_ +107 (c 0.001, MeOH); UV (MeOH) λ_max_ 214 nm (logε) (2.98) (Figure S24); m.p. 228–230 °C; IR (KBR) *v*_max_ 3729 broad (O-H), 3431(C=C-H), 2930 (C-H), and 1455 (C=C) cm^−1^ (Figure S25); CD nm [mdeg] 392 (−0.65), 382 (−1.89), 370 (−0.08), 356 (−2.95), 338 (−1.29), 324 (−4.01), 212 (52.15) (Figure S26); ^1^H NMR (CDCl_3_ 500 MHz) and ^13^C NMR (CDCl_3_ 150 MHz) data see Table 1; EI-MS *m*/*z* 442.4 [M]^+^ (Figure S22); HR-EI-MS (*m*/*z* 442.3832 [M]^+^ calcd. for C_30_H_48_O_2_ 442.3811) (Figure S23).

### 3.4. Gas Chromatography–Mass Spectrometry (GC-MS) Analysis

The hexane fraction was analyzed through Agilent 7000 GC/MS triple Quad, and Agilent 7890A GC system. The Agilent 7890A GC detector was used to accomplish the analysis. OPTIMA SN 23102-72 OPTIMA-5 was used to give temperature the maximum temperature during the analysis was 325 °C (30 m × 250 µm × 0.25 µm) and the phytocomponent were separated using helium as a carrier gas at a constant flow of 1.129 mL/min. A 2 µL volume of sample was injected, then analyzed by the Agilent 7000 triple quad mass detector. Initially, the temperature was maintained at 50 °C for 3 min, then it increased with 7 °C/min till 200 °C in 20 min, and then 7 °C/min till 300 °C in 25 min. Total runtime was 83.71 min. During this process, the injector temperature was maintained at 250 °C. Agilent 6890 gas chromatograph equipped with ZB-5MS (30 m × 0.32 ID and 0.25 μm film thickness) was combined with a Jeol, JMS-600H mass spectrometer operating in EI mode with ion source at 250 °C, and electron energy at 70 eV. Carrier gas volume was adjusted between 1.0 and 5.0 µL depending upon the detector response. The library used to identify the constituents was NIST Mass Spectral Search Program and Kovat’s retention indices.

## 4. Conclusions

Two undescribed ursane-type triterpenoids, named Polyanside A (**1**) and B (**2**), along with eleven known compounds (**3**-**13**), were isolated and elucidated from *Maranthes polyandra* (Benth.) Prance. The structures of these compounds were elucidated based on chemical evidence and multiple spectroscopic data. The hexane faction was analyzed by GC-MS, resulting the identification of forty-one compounds. The results contributed new knowledge to the phytochemistry of *M. polyandra.* Unfortunately, the tested compounds **1**, **2**, **5**, **6**, **8**, **11**, and **13** were found to be inactive on the anti-cancer and inflammatory assay. In addition, other compounds were not able to be employed for activity evaluation due to the poor quantity. The limited quantity of initial material presented difficulty in isolating more pure components or a greater quantity of obtained compounds. Further study with a sufficient quantity of initial material is required to discover potent molecules from this plant.

## Figures and Tables

**Table 1:** ^1^H NMR and ^13^C NMR data of compound **1** and **2**.

No.	1		2	
	*δ*_H_ *^a^*	*δ*_C_ *^b^*	*δ*_H_ *^a^*	*δ*_C_ *^b^*
1a	1.93 (o *^c^*)	41.7	1.63 (o)	40.9
1b	1.34 (o)		0.99 (o)	
2a	2.74 ddd (15.5, 13.5, 6.5)	34.5	1.63 (o)	27.4
2b	2.27 ddd (15.5, 5.0, 3.0)		1.61 (o)	
3	-	216.7	3.14 dd (10.0, 5.0)	79.1
4	-	48.7	-	39.6
5	1.22 br s	56.4	0.74 br s	55.5
6	4.49 br s	69.3	4.55 br s	68.7
7a	1.81 dd (14.5, 4.0)	40.8	1.79 dd (14.5, 4.0)	40.9
7b	1.55 (o)		1.52 (o)	
8	-	39.3	-	39.1
9	1.63 dd (11.5, 5.5)	47.3	1.56 dd(11.5, 6.0)	48.0
10	-	36.3	-	36.3
11a	2.11 ddd (18.0, 11.5, 3.0)	23.5	2.05 ddd (18.0, 12.0, 3.0)	23.3
11b	1.99 (o)		1.95 (o)	
12	5.18 dd (5.0, 3.0)	124.5	5.16 dd (4.5, 3.0)	124.8
13	-	139.0	-	138.7
14	-	42.8	-	42.7
15a	1.89 (o)	26.6	1.87 (o)	26.6
15b	0.97 ddd (13.0, 4.0, 2.0)		0.96 (o)	
16a	1.98 (o)	28.0	1.98 (o)	28.1
16b	0.86 (o)		0.87 (o)	
17	-	33.8	-	33.8
18	1.33 (o)	59.1	1.32 (o)	59.1
19	1.31 (o)	39.7	1.32 (o)	39.7
20	0.88 (o)	39.6	0.87 (o)	39.6
21a	1.37 (o)	31.2	1.37 (o)	31.3
21b	1.24 (o)		1.23 (o)	
22a	1.41 (o)	41.5	1.40 (o)	41.5
22b	1.28 (o)		1.28 (o)	
23	1.15 s	26.0	1.06 s	28.0
24	1.40 s	23.9	1.16 s	17.2
25	1.50 s	16.7	1.32 s	17.0
26	1.35 s	18.9	1.28 s	18.6
27	1.02 s	23.3	1.02 s	23.4
28	0.80 s	28.7	0.79 s	28.7
29	0.78 d (6.0)	17.4	0.78 d (6.0)	17.4
30	0.90 br s	21.4	0.90 br s	21.4

*^a^* measured in CDCl_3_ at 500 MHz. *^b^* measured in CDCl_3_ at 125 MHz. *^c^* o = overlapped.

**Table 2:** Chemical constituents obtained from GC-MS analysis of Hexane fraction of *M. polyandra*.

Peak Number	RT (min)	Compound Name	Molecular Formula	Molecular Weight	Area Sum%	Compound Nature	Uses	References
1.	6.36	2,4-Dimethylhexane	C_8_H_18_	114	0.1	Hydrocarbon	Flavor	[37]
2.	10.47	2-Heptenal	C_7_H_12_O	112	0.13	Aldehyde	Flavor	[37]
3.	12.28	2-Ethylhexanol	C_8_H_18_O	130	0.49	Alcohol	Dispersants, printing, dying, and paints	[38]
4.	12.57	*N-*Methyl-2-pyrrolidone	C_5_H_9_NO	99	0.52	Lactam	Recover certain hydrocarbons generated in processing of petrochemicals	[39]
5.	13.00	2-Octenal	C_8_H_14_O	126	0.06	Aldehyde	-	
6.	14.07	*n-*Nonanal	C_9_H_18_O	142	0.04	Aldehyde	-	
7.	14.49	Methyl caprylate	C_9_H_18_O_2_	158	0.06	Ester	-	
8.	15.57	Caprylic acid	C_8_H_16_O_2_	144	0.44	Fatty acid	-	
9.	17.55	2-Decenol	C_10_H_18_O	154	0.42	Aldehyde	Flavor	[37]
10.	17.59	Nonanoic acid	C_9_H_18_O_2_	158	0.02	Aldehyde	Flavor	[37]
11.	18.24	2,4-Decadienal	C_9_H_18_O_2_	158	0.13	Fatty acid	Flavor	[37]
12.	18.71	2,4-Decanedienal	C_10_H_16_O	152	0.17	Aldehyde	Flavor	[37]
13.	19.54	*n-*Decanoic acid	C_10_H_20_O_2_	172	0.13	Fatty acid	-	
14.	20.46	3-Hydroxy-4-methoxybenzaldehyde acetate	C_10_H_10_O_4_	194	1.16	Aromatic compound	Flavor	[37]
15.	22.60	Vanillic acid methyl ester	C_9_H_10_O_4_	182	0.06	Aromatic compound	Flavor	[37]
16.	23.90	Methyl 4,7,10,13-hexadecatetraenoate	C_17_H_26_O_2_	262	0.35	Fatty ester	-	
17.	25.55	*n-*heptadecane	C_17_H_36_	240	0.1	Alkane	-	
18.	27.73	*n-*octadecane	C_18_H_38_	254	0.14	Hydrocarbon	A volatile oil	[37]
19.	30.08	1-hexadecanol	C_16_H_34_O	242	2.15	Alcohol	-	
20.	30.66	*n-*Nonadecane	C_19_H_40_	268	0.86	Hydrocarbon	-	
21.	31.62	*n-*Hexadecanoic acid methyl ester	C_17_H_34_O_2_	270	5.17	Ester	-	
22.	33.5	*n-*Hexadecanoic acid	C_16_H_32_O_2_	256	0.73	Hydrocarbon	-	
23.	34.71	Eicosane	C_20_H_42_	282	0.93	Hydrocarbon	Used for the treatment of eczema	[40]
24.	37.98	9-Octadece*n-*1-ol	C_18_H_36_O	268	2.36	Alcohol	-	
25.	39.39	1-Heptadecanol	C_17_H_36_O	256	1.24	Alcohol	-	
26.	40.12	Methyl linoleate	C_19_H_34_O_2_	294	2.07	Fatty	Anti-inflammatory	[37]
27.	40.54	Methyl (10E)-10-octadecenoat	C_19_H_36_O_2_	296	2.61	Ester	-	
28.	40.88	Oleic acid methyl ester	C_19_H_36_O_2_	296	0.39	Ester	-	
29.	42.25	*n-*Octadecanoic acid, methyl ester	C_20_H_40_O_2_	312	0.74	Alcohol	Emulsifier	[37]
30.	47.12	Eicosanol	C_20_H_40_O_2_	298	0.63	Arachidyl alcohol	Emollient and thickener	[37]
31.	49.08	Kauran-16-ol	C_20_H_34_O	290	0.7	Diterpene	-	
32.	55.31	Methyl docosanoate	C_23_H_46_O_2_	354	0.62	Ester	-	
33.	67.96	Stigmasterol	C_29_H_48_O	412	6.22	Sterol	Anti-inflammatory, antipyretic, antiarthritic, anti-ulcer, insuli*n-*releasing, and estrogenic effects	[34,41,42]
34.	69.53	γ-Sitosterol	C_29_H_50_O	414	2.99	Sterol	Antidiabetic activity	[43]
35.	71.44	β-amyrone	C_30_H_50_O	426	1.42	Triterpene	Anti-inflammatory activity	[41,44]
36.	71.88	4,22-Stigmastadiene-3-one	C_29_H_46_O	410	8.33	Steroid	Antimicrobial activity	[41]
37.	73.91	Stigmast-4-*en-*3-one	C_29_H_48_O	412	7.4	Sterol	Hypoglycemic activity	[45]
38.	77.21	Friedelan-3-one	C_30_H_50_O	426	0.92	Triterpene	Antimicrobial activity	[46]
39.	77.81	3-Methoxystigmasta-5,22-diene	C_30_H_50_O	426	3.85	Steroid	-	
40.	79.82	β-Amyrin methyl ether	C_31_H_52_O	440	7.71	Pentacyclic triterpene	-	
41.	80.49	5α-Stigmastane-3,6-dione	C_29_H_48_O_2_	428	3.27	Sterol	-	

## Data Availability

Not applicable.

## Supplementary Materials

The following supporting information can be downloaded online. S3.5 Anti-cancer and cytotoxicity assay. S3.6 Nitric oxide (NO) inhibition assay. Figure S1. EI-MS spectrum of compound **1**. Figure S2. HR-EI-MS spectrum of compound **1**. Figure S3. UV spectrum of compound **1**. Figure S4. IR spectrum of compound **1**. Figure S5. CD spectrum of compound **1**. Figure S6. ^1^H NMR spectrum of compound **1** (500 MHz, CDCl3). Figure S7. ^1^H NMR assignment-1 of compound **1**. Figure S8. ^1^H NMR assignment-2 of compound **1**. Figure S9. ^13^C NMR spectrum of compound **1** (150 MHz, CDCl3). Figure S10. ^13^C NMR assignment-1 of compound **1**. Figure S11. ^13^C NMR assignment-2 of compound **1**. Figure S12. ^13^C NMR assignment-3 of compound **1**. Figure S13. DEPT spectrum of compound **1**. Figure S14. ^1^H–^1^H COSY spectrum-1 of compound **1**. Figure S15. ^1^H–^1^H COSY spectrum-2 of compound **1**. Figure S16. HSQC spectrum-1 of compound **1**. Figure S17. HSQC spectrum-2 of compound **1**. Figure S18. HMBC spectrum-1 of compound **1**. Figure S19. HMBC spectrum-2 of compound **1**. Figure S20. NOESY spectrum-1 of compound **1**. Figure S21. NOESY spectrum-2 of compound **1**. Figure S22. EI-MS spectrum of compound **2**. Figure S23. HR-EI-MS spectrum of compound **2**. Figure S24. UV spectrum of compound **2**. Figure S25. IR spectrum of compound **2**. Figure S26. CD spectrum of compound **2**. Figure S27. ^1^H NMR spectrum of compound **2** (500 MHz, CDCl3). Figure S28. ^1^H NMR assignment-1 of compound **2**. Figure S29. ^1^H NMR assignment-2 of compound **2**. Figure S30. ^13^C NMR spectrum of compound **2** (150 MHz, CDCl3). Figure S31. ^13^C NMR assignment-1 of compound **2**. Figure S32. ^13^C NMR assignment-2 of compound **2**. Figure S33. ^13^C NMR assignment-3 of compound **2**. Figure S34. ^1^H–^1^H COSY spectrum-1 of compound **2**. Figure S35. ^1^H–^1^H COSY spectrum-2 of compound **2**. Figure S36. HSQC spectrum-1 of compound **2**. Figure S37. HSQC spectrum-2 of compound **2**. Figure S38. HMBC spectrum-1 of compound **2**. Figure S39. HMBC spectrum-2 of compound **2**. Figure S40. NOESY spectrum-1 of compound **2**. Figure S41. NOESY spectrum-2 of compound **2**.

## Sample Availability

Samples of the compounds are available from the authors.
