# Supplementary material for: Phytochemical Characterizations of Maranthes polyandra (Benth.) Prance
Source: Molecules. 2022 Feb 15;27(4):1316. doi: 10.3390/molecules27041316 (PMC8879441; doi:10.3390/molecules27041316)
Supplement: Supplementary file 1 [file molecules-27-01316-s001.zip › molecules-1559745-supplementary.pdf]

Supplementary data

Phytochemical characterizations of *Maranthes polyandra* (Benth.) Prance

Nida Ali [1,4], Farooq-Ahmad Khan [1,8], Kayode Muritala Salawu [3], Rimsha Irshad [1], Almas Jabeen [7], Chunlei Zhang [6], M. Iqbal Choudhary [1,4,7], Xinmin Liu [2,5,*], Yan Wang [1,4,*]

[1] H. E. J. Research Institute of Chemistry, International Center for Chemical and Biological Sciences, University of Karachi, Karachi 75270, Pakistan; s.nidaali.m@gmail.com (N.A.); farooq.khan@iccs.edu (F.A.K); iqbal.choudhary@iccs.edu (M.I.C); yanwang@iccs.edu (Y.W.).

[2].Sino-Pakistan Cooperation Center for Traditional Chinese Medicine, Hunan University of Medicine, Huaihua 418000, Hunan, China; liuxinmin@hotmail.com (X.M.L.),

[3] Department of Pharmacognosy and Drug Development, University of Ilorin, Nigeria; pharmmks@yahoo.com (K.M.S.)

[4] Sino-Pakistan Cooperation Center for Traditional Chinese Medicine, International Center for Chemical and Biological Sciences, University of Karachi, Karachi 75270, Pakistan

[5] Institute of Medicinal Plant Development, Chinese Academy of Medical Sciences, Beijing 100193, China

[6] School of Traditional Chinese Pharmacy, China Pharmaceutical University, Nanjing, Jiangsu 211198, China; zhangchunlei11@sina.com (C.L.Z.)

[7] Dr. Panjwani Center for Molecular Medicine and Drug Research, International Center for Chemical and Biological Sciences, University of Karachi, Karachi 75270, Pakistan

[8] Third World Center (TWC) for Chemical Sciences, International Center for Chemical & Biological Sciences, University of Karachi-75270, Pakistan

*Correspondence: yanwang@iccs.edu (Y.W.); liuxinmin@hotmail.com (X.M.L.)

Table of Contents

S3.5 Cytotoxicity assay against cancer and normal cell lines....4

Table S1 Experimental data of Cytotoxicity assay against cancer and normal cell lines....4

S3.6 Nitric Oxide (NO) production inhibition assay....4

Table S2. Experimental data of Nitric Oxide (NO) production inhibition assay....5

Figure S1. EI-MS spectrum of compound **1**....5

Figure S2. HR-EI-MS spectrum of compound **1**....6

Figure S3. UV spectrum of compound **1**....7

Figure S4. IR spectrum of compound **1**....8

Figure S5. CD spectrum of compound **1**....9

Figure S6. ^{1}H NMR spectrum of compound **1** (500 MHz, $CDCl_3$)....10

Figure S7. ^{1}H NMR assignment-1 of compound **1**....10

Figure S8. ^{1}H NMR assignment-2 of compound **1**....11

Figure S9. ^{13}C NMR spectrum of compound **1** (150 MHz, $CDCl_3$)....11

Figure S10. ^{13}C NMR assignment-1 of compound **1**....12

Figure S11. ^{13}C NMR assignment-2 of compound **1**....12

Figure S12. ^{13}C NMR assignment-3 of compound **1**....13

Figure S13. DEPT spectrum of compound **1**....13

Figure S14. ^{1}H–^{1}H COSY spectrum-1 of compound **1**....14

Figure S15. ^{1}H–^{1}H COSY spectrum-2 of compound **1**....14

Figure S16. HSQC spectrum-1 of compound **1**....15

Figure S17. HSQC spectrum-2 of compound **1**....15

Figure S18. HMBC spectrum-1 of compound **1**....16

Figure S19. HMBC spectrum-2 of compound **1**....16

Figure S20. NOESY spectrum-1 of compound **1**....17

Figure S21. NOESY spectrum-2 of compound **1**....17

Figure S22. EI-MS spectrum of compound **2**....18

Figure S23. HR-EI-MS spectrum of compound **2**....19

Figure S24. UV spectrum of compound **2**....20

Figure S25. IR spectrum of compound **2**....21

Figure S26. CD spectrum of compound **2**....22

Figure S27. ^{1}H NMR spectrum of compound **2** (500 MHz, $CDCl_3$)....23

Figure S28. ^{1}H NMR assignment-1 of compound **2**....23

Figure S29. ^{1}H NMR assignment-2 of compound **2**....24

Figure S30. ^{13}C NMR spectrum of compound **2** (150 MHz, $CDCl_3$). 24
Figure S31. ^{13}C NMR assignment-1 of compound **2**. 25
Figure S32. ^{13}C NMR assignment-2 of compound **2**. 25
Figure S33. ^{13}C NMR assignment-3 of compound **2**. 26
Figure S34. ^{1}H–^{1}H COSY spectrum-1 of compound **2**. 26
Figure S35. ^{1}H–^{1}H COSY spectrum-2 of compound **2**. 27
Figure S36. HSQC spectrum-1 of compound **2**. 27
Figure S37. HSQC spectrum-2 of compound **2**. 28
Figure S38. HMBC spectrum-1 of compound **2**. 28
Figure S39. HMBC spectrum-2 of compound **2**. 29
Figure S40. NOESY spectrum-1 of compound **2**..29
Figure S41. NOESY spectrum-2 of compound **2**..30

S3.5 Cytotoxicity assay against cancer and normal cell lines

The human cancerous cell lines MCF-7 (breast cancer), NCI-H460 (Lung cancer), Hela (Cervical cancer) and Human normal cell BJ were collected from cell culture biobank (PCMD, ICCBS) obtained from American Type Culture Collection (ATCC). They were cultured in DMEM medium (for MCF-7, Hela, and BJ) and RPMI (ATCC modified medium for NCI-H460) supplemented with 5% FBS, 100 IU/mL of penicillin, and 100 µg/mL of streptomycin, and kept at 37°C in 5% CO_2 incubator. The inhibition of cancerous cells was investigated with the help of 3-[4, 5-dimethylthiazole-2-yl]-2, 5-diphenyl-tetrazolium bromide (MTT) assay. For this, we plated 100 µL/well of cell solution (10×10^4 cells/mL MCF-7 cells), (5×10^4 cells/mL NCI-H460 cells), (6×10^4 cells/mL Hela cells), were added into 96-well plate and incubated for 24 hrs at 37°C, before treatment with 6.25-50 µM of positive control Doxorubicin, compounds **1**, **2**, **5**, **6**, **8**, **11**, and **13,** or negative control DMSO for 48 hrs. After this, 20 µL of MTT (5 mg/mL), the dye was added to each well and the plates were incubated for 4 h at 37°C. After the completion of incubation of 100 µL, DMSO was added to each well to dissolve the purple formazan crystal and the absorbance was taken at 570 nm using an ELISA plate reader. The growth inhibition of cancer cells was expressed as IC_{50} value.

Table S1 Experimental data of Cytotoxicity assay against cancer and normal cell lines

No.	MCF7<br>% inhibition (50 µM)	H460<br>% inhibition (50 µM)	Hela<br>% inhibition (50 µM)	BJ<br>% inhibition (50 µM)
**1**	1.1	7.5	3.9	2.9
**2**	2.0	8.2	7.9	6.6
**5**	7.5	2.8	3.7	3.9
**6**	5.3	3.4	11.7	8.4
**8**	1.2	2.6	2.0	3.8
**11**	9.6	6.9	8.6	2.8
**13**	1.0	6.2	8.8	9.3
**Doxorubicin**	85.3<br>IC_{50} 2.3 ± 0.2 µM	85.3<br>IC_{50} 1.4 ± 0.1 µM	99.5<br>IC_{50} 0.29 ± 0.01 µM	

S3.6 Nitric Oxide (NO) production inhibition assay

The J774.2 cell line (mouse macrophage) was cultivated in 75 cc flasks IWAKI (Japan), containing DMEM and FBS (10%), and were incubated in CO_2 (5%) at 37°C. Fully-grown

cells (10^6 cells/mL) were transferred into the 96-well plate, and nitrite production was induced by by *E. coli* (30 µg/mL) LPS (lipopolysaccharide). Compounds **1**, **2**, **5**, **6**, **8**, **11**, and **13**, positive control L-NMMA**,** and negative control DMSO were tested with different concentrations (1, 10, and 25 µg/mL), and placed in CO_2 (5%) at 37°C. Griess method was used to analyze accumulation of nitrite in the cells (J774.2).

Table S2. Experimental data of Nitric Oxide (NO) production inhibition assay

No.	IC_{50} (µg/mL)
**1**	> 100
**2**	> 100
**5**	> 100
**6**	> 100
**8**	> 100
**11**	> 100
**13**	> 100
LNMMA	24.2 ± 0.8

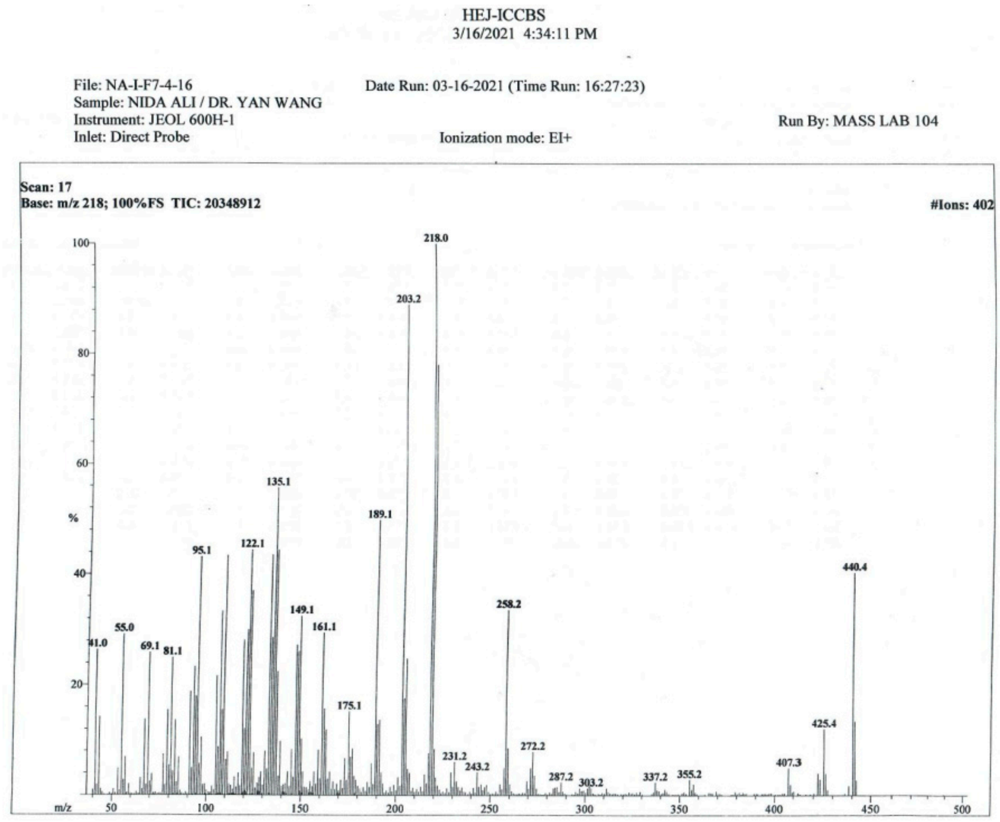

Figure S1. EI-MS spectrum of compound **1**.

Mass	Relative Intensity	Theoretical Mass	Delta [ppm]	Delta [mmu]	RDB	Composition
259.2351	2.0	259.2426	-29.0	-7.5	4.5	$C_{19} H_{31}$
		259.2273	29.9	7.7	0.5	$C_{15} H_{31} O_{3}$
264.2491	5.8	264.2453	14.3	3.8	3.0	$C_{18} H_{32} O_{1}$
265.2482	6.9	265.2531	-18.6	-4.9	2.5	$C_{18} H_{33} O_{1}$
266.2644	1.0	266.2610	12.8	3.4	2.0	$C_{18} H_{34} O_{1}$
269.2202	1.4	269.2269	-25.1	-6.8	6.5	$C_{20} H_{29}$
		269.2117	31.6	8.5	2.5	$C_{16} H_{29} O_{3}$
271.2415	1.9	271.2426	-4.1	-1.1	5.5	$C_{20} H_{31}$
272.2485	2.5	272.2504	-6.9	-1.9	5.0	$C_{20} H_{32}$
273.2364	3.0	273.2430	-24.2	-6.6	0.5	$C_{16} H_{33} O_{3}$
279.2297	1.4	279.2324	-9.7	-2.7	3.5	$C_{18} H_{31} O_{2}$
280.2426	1.3	280.2402	8.5	2.4	3.0	$C_{18} H_{32} O_{2}$
281.2563	2.1	281.2481	29.2	8.2	2.5	$C_{18} H_{33} O_{2}$
282.2584	1.2	282.2559	8.8	2.5	2.0	$C_{18} H_{34} O_{2}$
283.2573	2.6	283.2637	-22.6	-6.4	1.5	$C_{18} H_{35} O_{2}$
284.2456	1.3	284.2504	-16.8	-4.8	6.0	$C_{21} H_{32}$
		284.2351	36.8	10.5	2.0	$C_{17} H_{32} O_{3}$
299.2464	1.2	299.2375	29.9	9.0	6.5	$C_{21} H_{31} O_{1}$
327.2931	2.4	327.2899	9.6	3.1	1.5	$C_{20} H_{39} O_{3}$
		327.3052	-37.0	-12.1	5.5	$C_{24} H_{39}$
407.3272	1.7	407.3314	-10.2	-4.1	8.5	$C_{29} H_{43} O_{1}$
		407.3161	27.3	11.1	4.5	$C_{25} H_{43} O_{4}$
422.3499	3.3	422.3549	-11.8	-5.0	8.0	$C_{30} H_{46} O_{1}$
		422.3396	24.4	10.3	4.0	$C_{26} H_{46} O_{4}$
423.3334	1.8	423.3263	16.8	7.1	8.5	$C_{29} H_{43} O_{2}$
		423.3474	-33.1	-14.0	3.5	$C_{26} H_{47} O_{4}$
425.3376	2.2	425.3420	-10.3	-4.4	7.5	$C_{29} H_{45} O_{2}$
		425.3267	25.6	10.9	3.5	$C_{25} H_{45} O_{5}$
		425.3208	39.4	16.8	12.5	$C_{32} H_{41}$
438.3495	1.1	438.3498	-0.6	-0.3	8.0	$C_{30} H_{46} O_{2}$
		438.3345	34.2	15.0	4.0	$C_{26} H_{46} O_{5}$
440.3648	7.9	440.3654	-1.4	-0.6	7.0	$C_{30} H_{48} O_{2}$
		440.3502	33.3	14.7	3.0	$C_{26} H_{48} O_{5}$
441.3616	3.6	441.3580	8.2	3.6	2.5	$C_{26} H_{49} O_{5}$
		441.3521	21.5	9.5	11.5	$C_{33} H_{45}$
		441.3733	-26.4	-11.6	6.5	$C_{30} H_{49} O_{2}$

Figure S2. HR-EI-MS spectrum of compound **1**.

THERMO ELECTRON ~ VISIONpro SOFTWARE V4.10

Operator Name	Zainab Rizvi	Date of Report	8/31/2021
Department	Analytical Lab,. Nanotechnology Cntr.	Time of Report	2:49:32PM
Organization	ICCBS ,University of Kar.PAKISTAN		
Information	Aamir/Dr.Yan		

Scan Graph

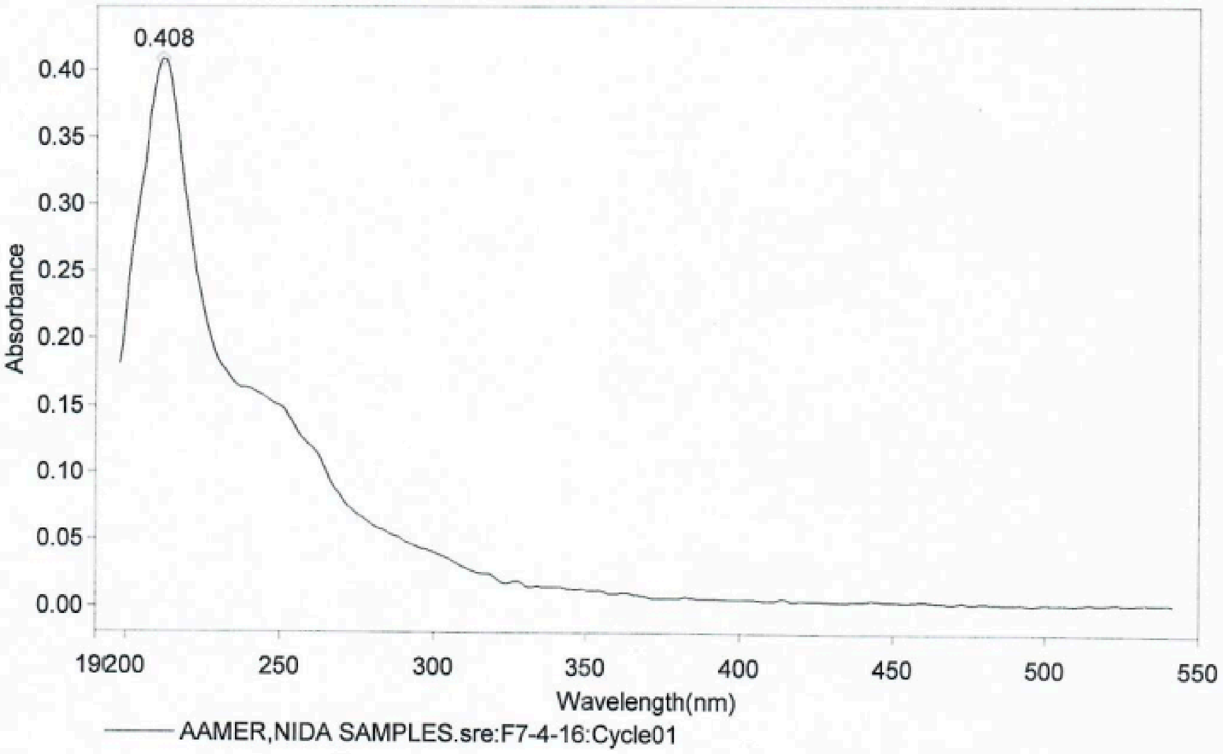

Results Table - AAMER,NIDA SAMPLES.sre,F7-4-16,Cycle01

nm	A	**Peak Pick Method**
212.00	0.408	Find 8 Peaks Above -3.0000 A
		Start Wavelength 190.00 nm
		Stop Wavelength 550.00 nm
		Sort By Wavelength

Sensitivity Auto

Figure S3. UV spectrum of compound **1**.

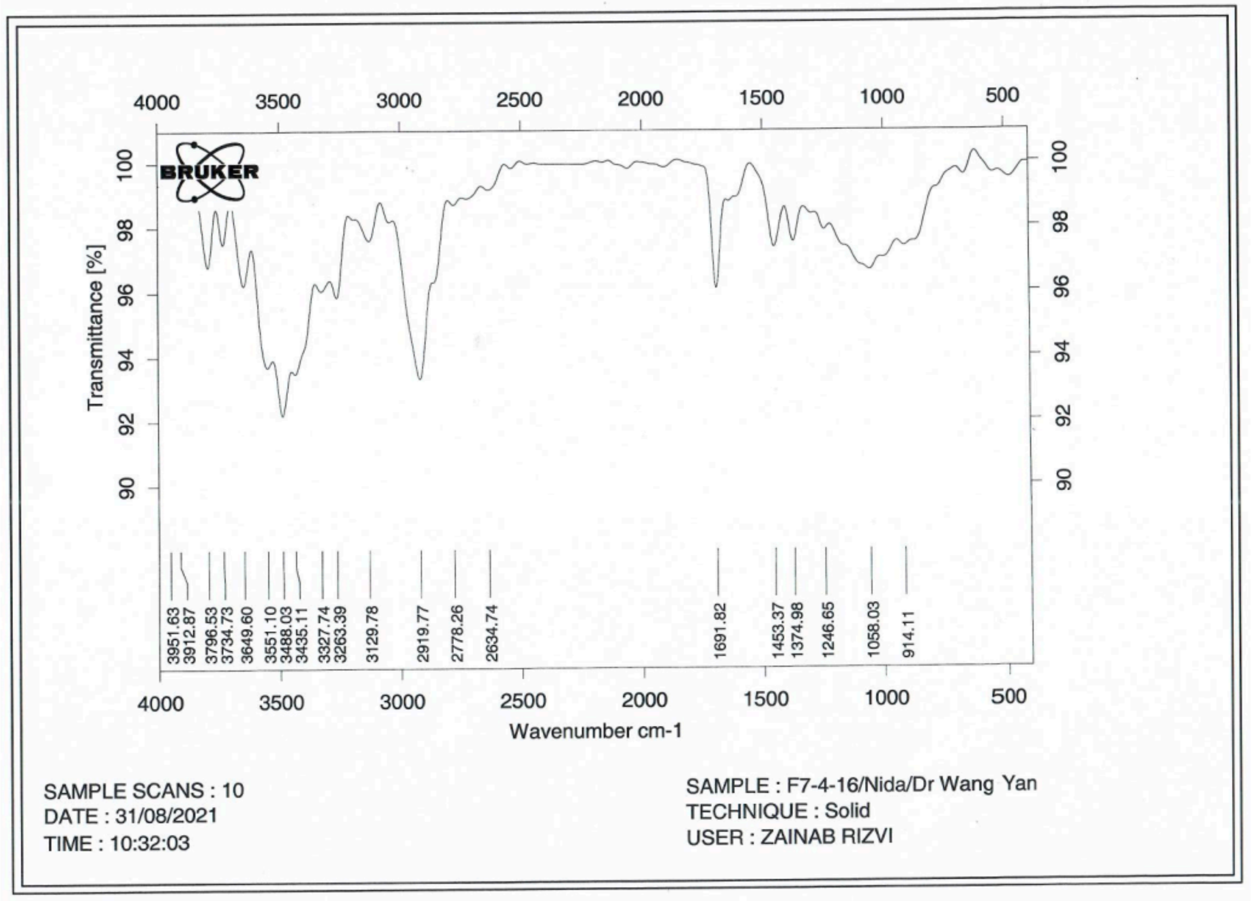

Figure S4. IR spectrum of compound **1**.

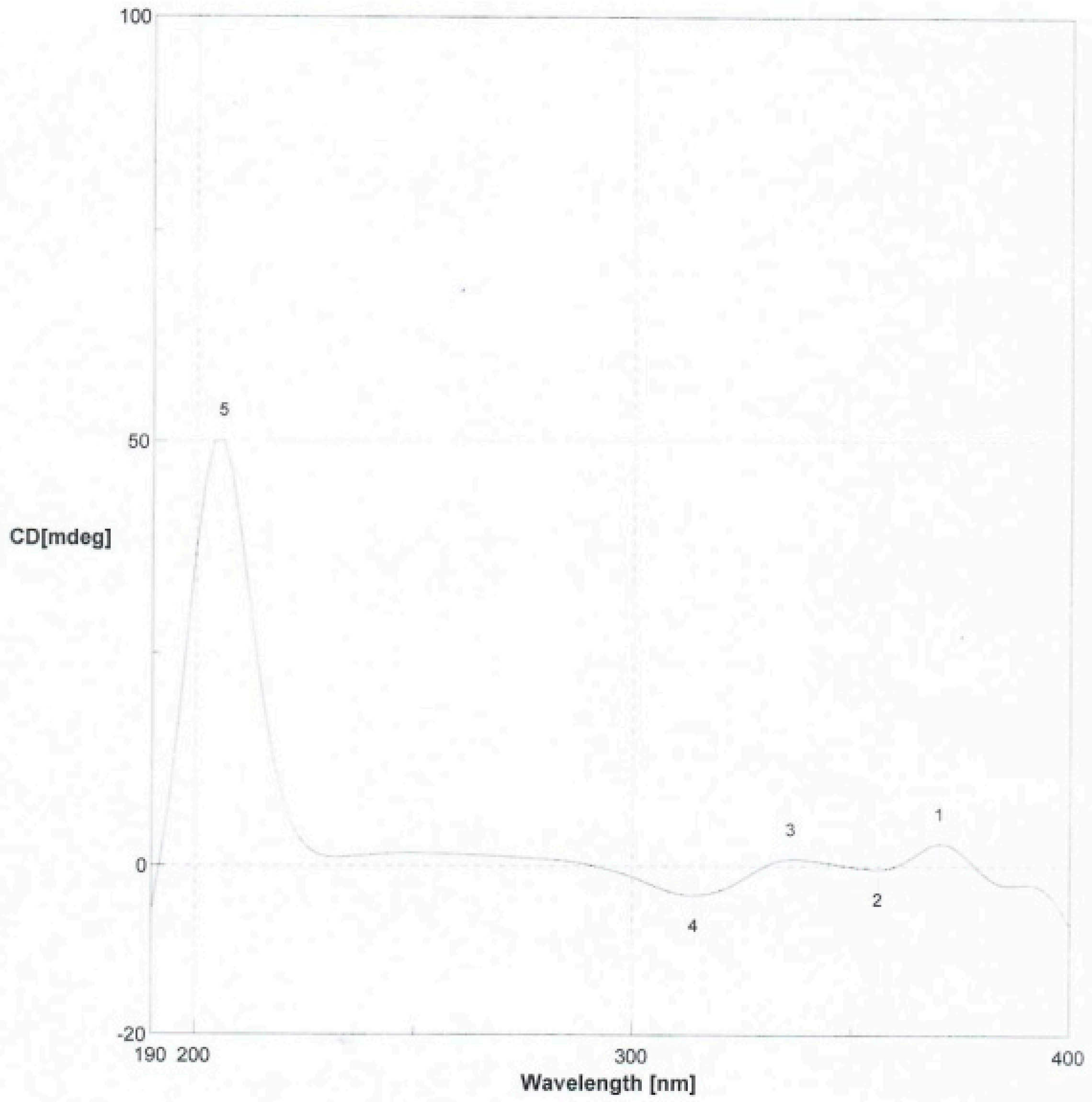

Date/Time	8/30/2021 11:06AM
Operator	ZAINAB RIZVI
File Name	F7-4-16 final
Sample Name	F7-4-16
Comment	Nida/Dr.Yan

No.	nm	CD[mdeg]	No.	nm	CD[mdeg]	No.	nm	CD[mdeg]
1	370	2.6674	2	356	-0.365594	3	336	0.802895
4	314	-3.46305	5	206	50.1367			

Figure S5. CD spectrum of compound **1**.

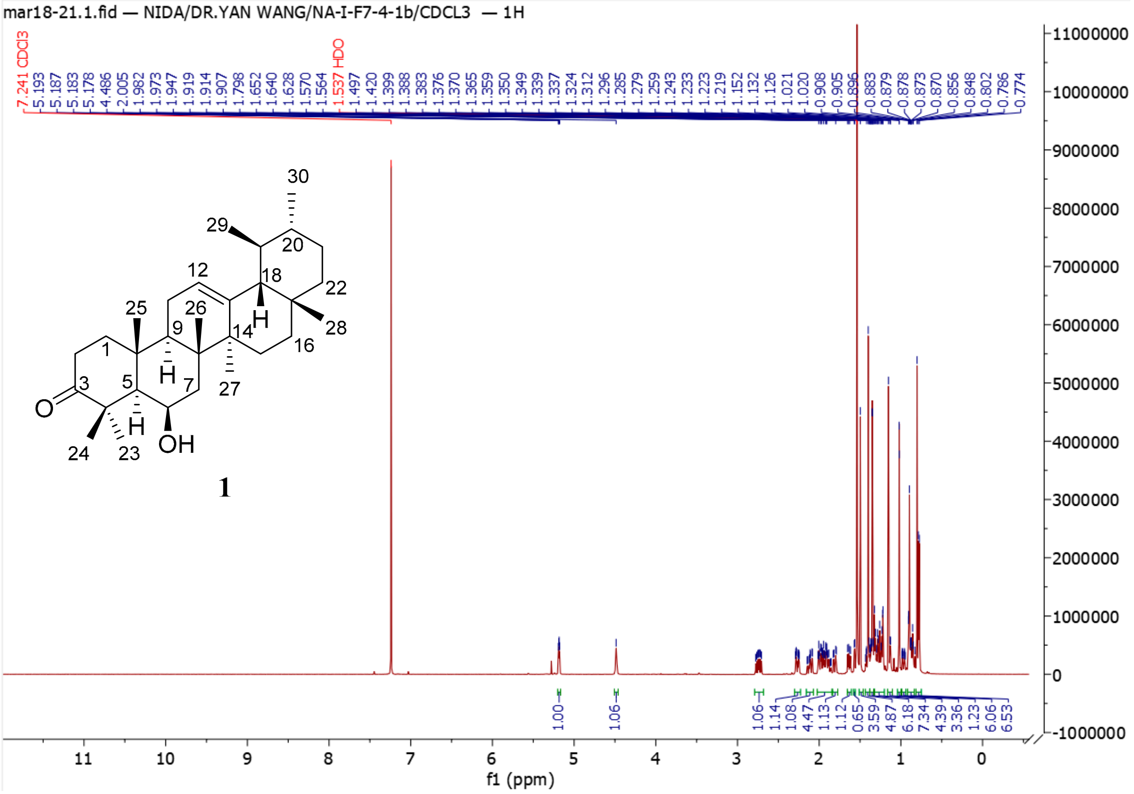

Figure S6. ^{1}H NMR spectrum of compound **1** (500 MHz, $CDCl_3$).

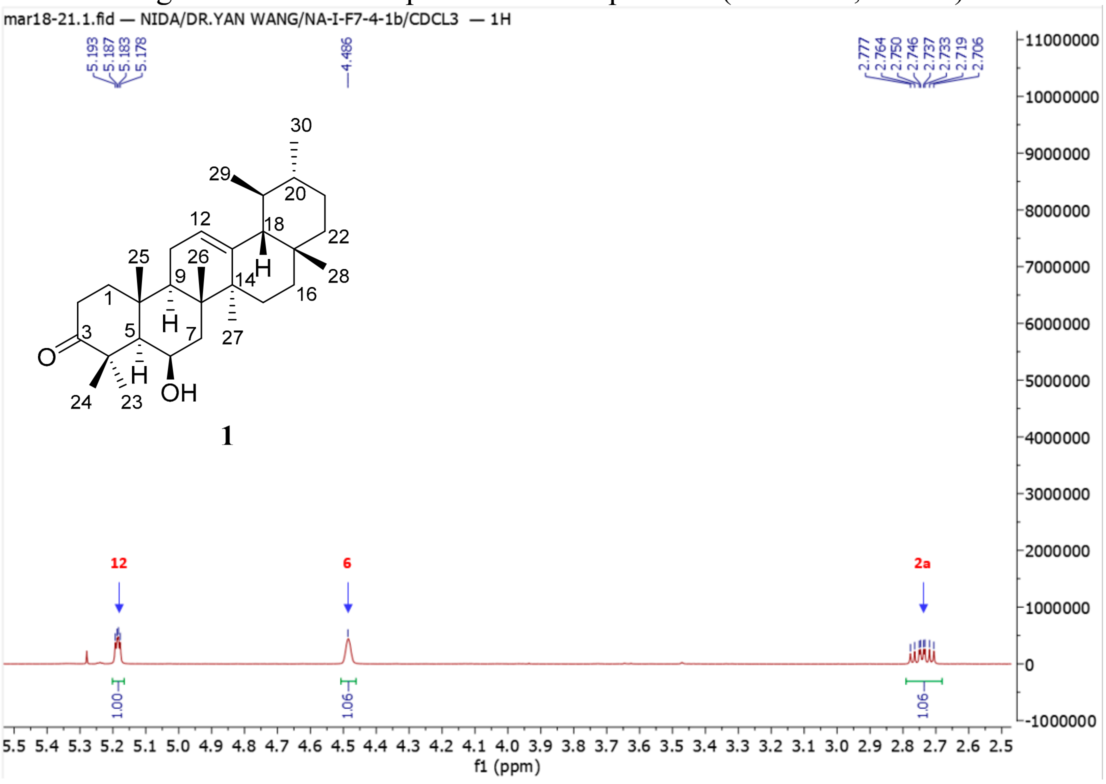

Figure S7. ^{1}H NMR assignment-1 of compound **1**.

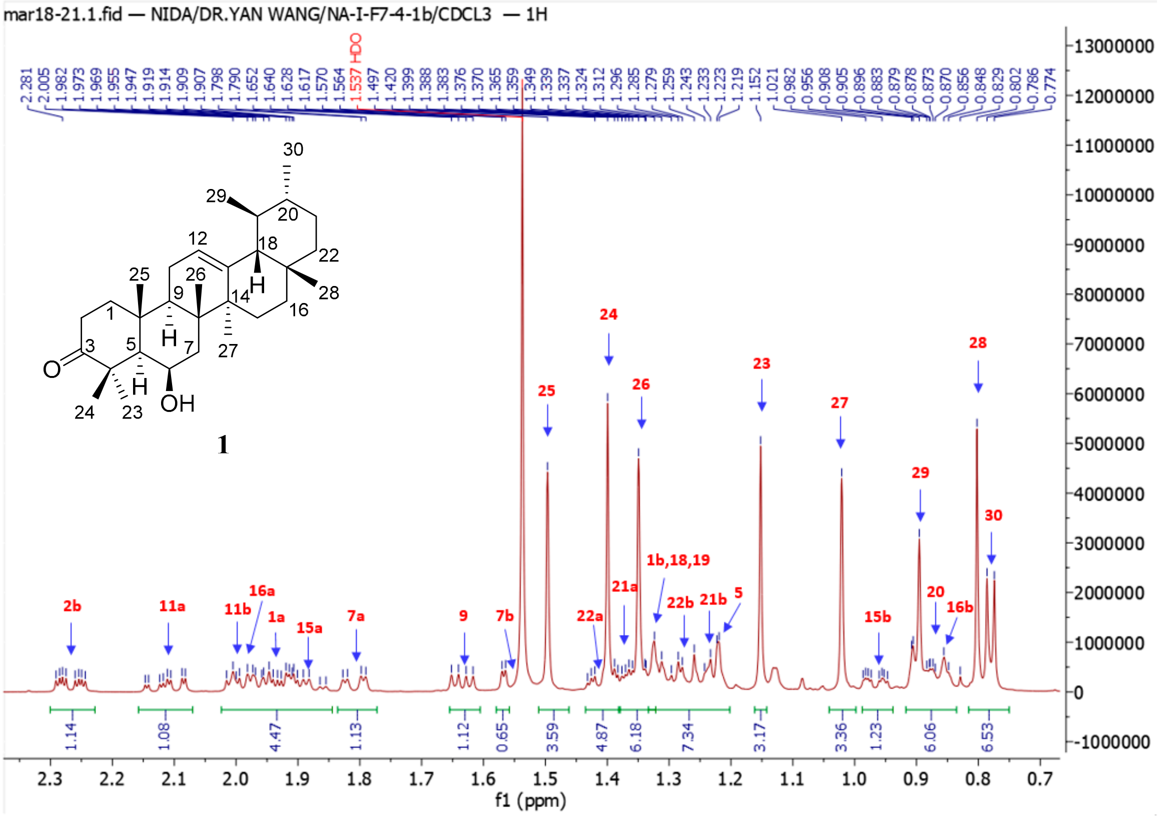

Figure S8. ^{1}H NMR assignment-2 of compound **1**.

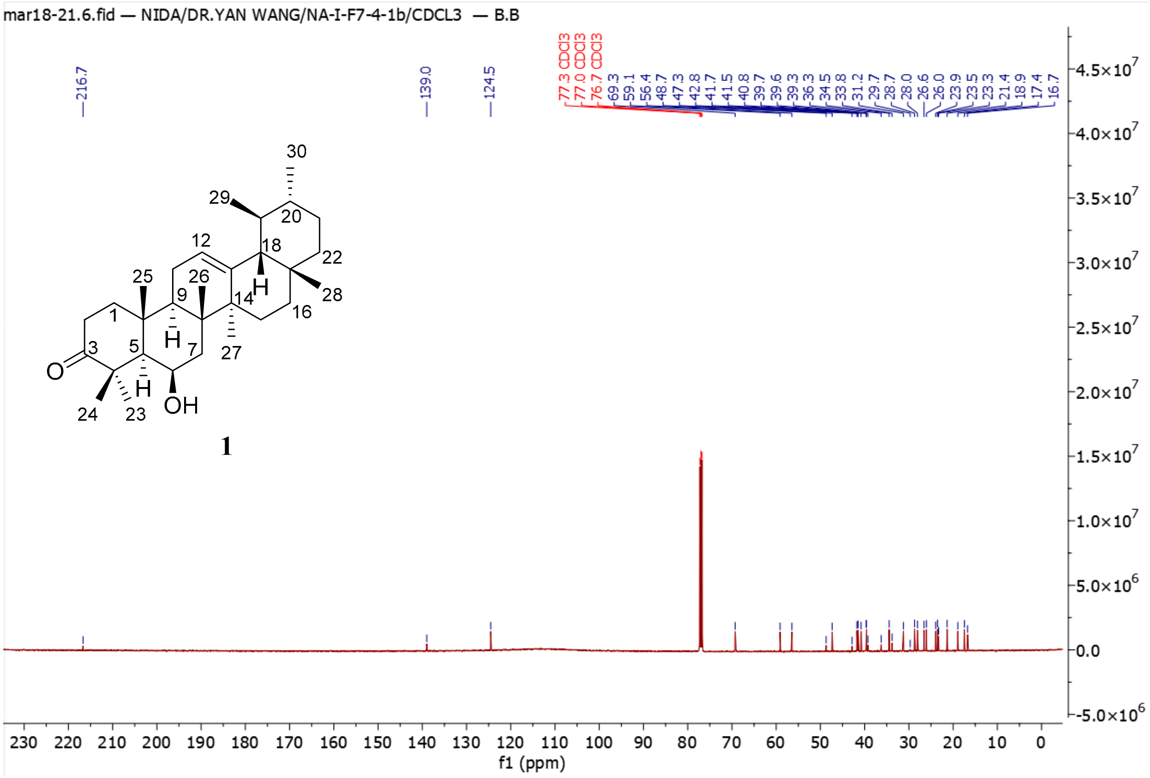

Figure S9. ^{13}C NMR spectrum of compound **1** (150 MHz, $CDCl_3$).

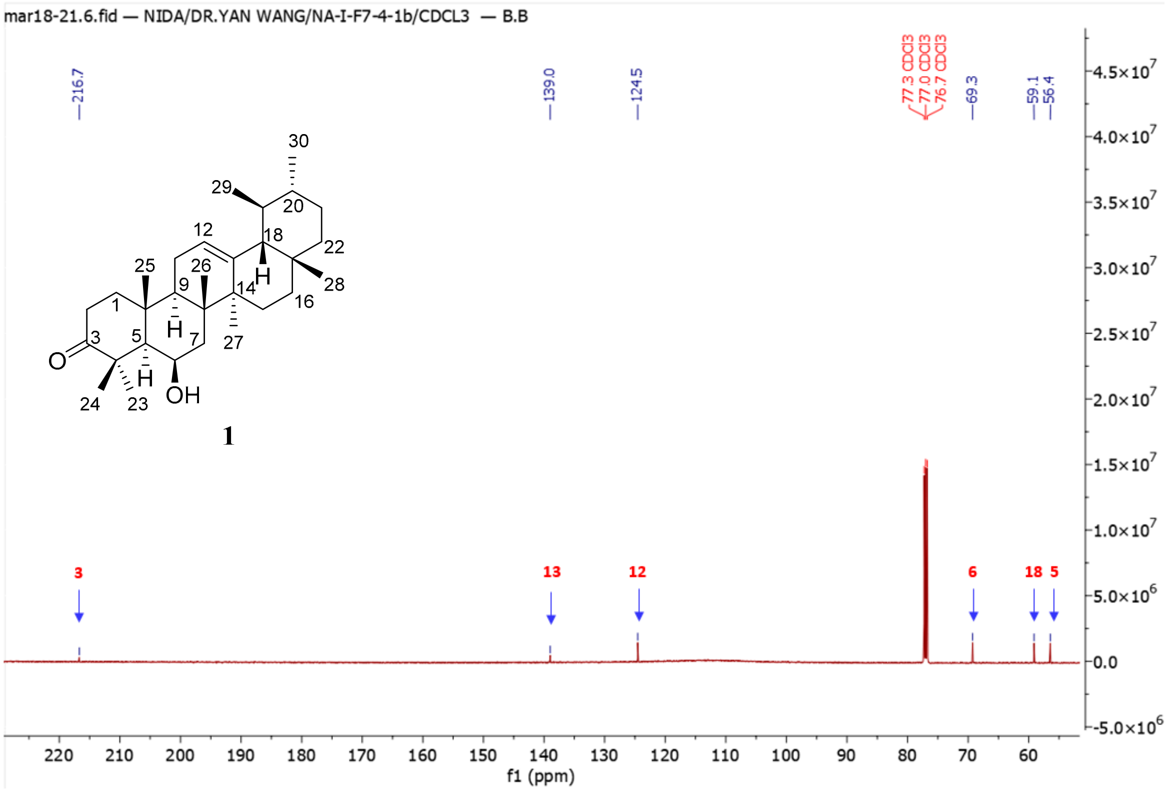

Figure S10. ^{13}C NMR assignment-1 of compound **1**.

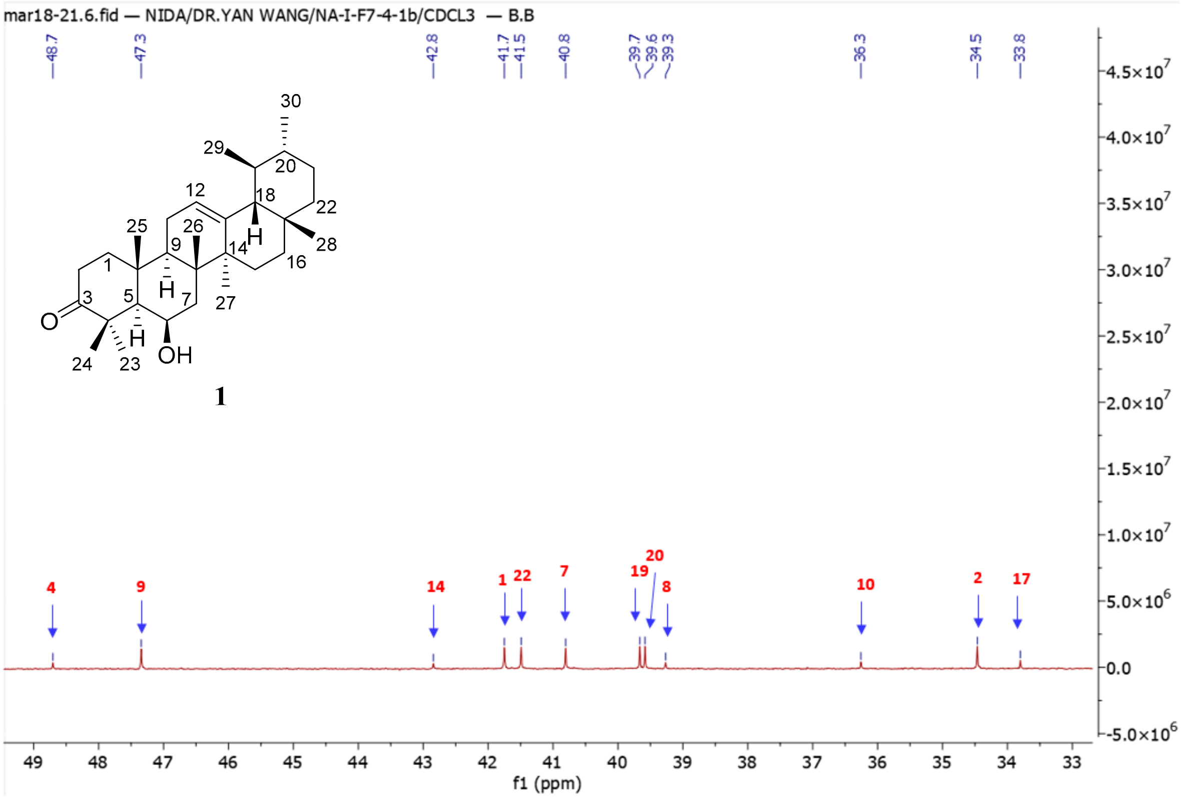

Figure S11. ^{13}C NMR assignment-2 of compound **1**.

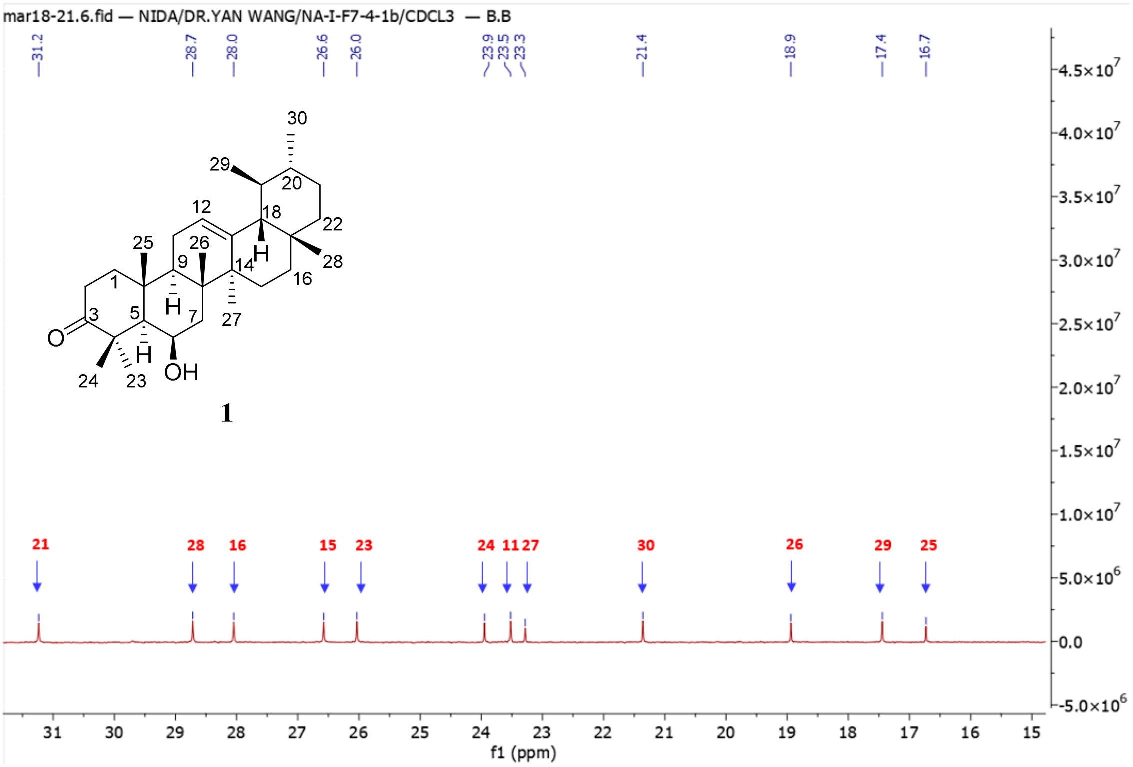

Figure S12. ^{13}C NMR assignment-3 of compound **1**.

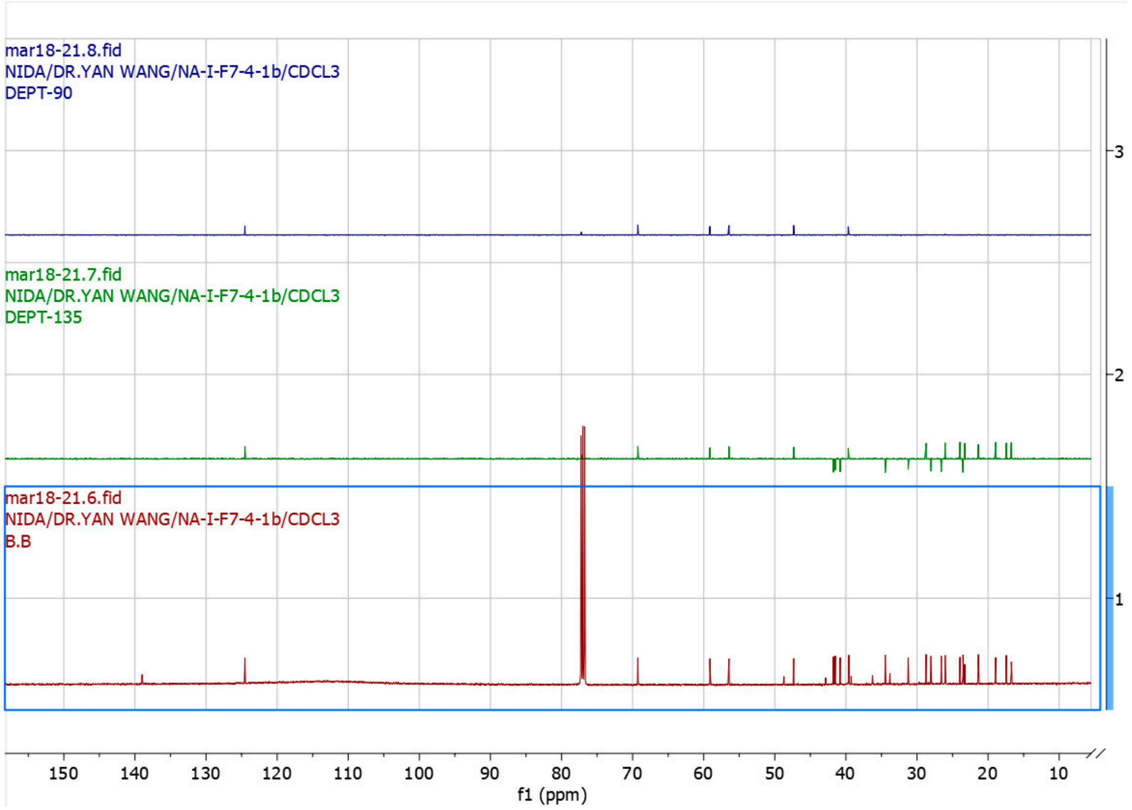

Figure S13. DEPT spectrum of compound **1**.

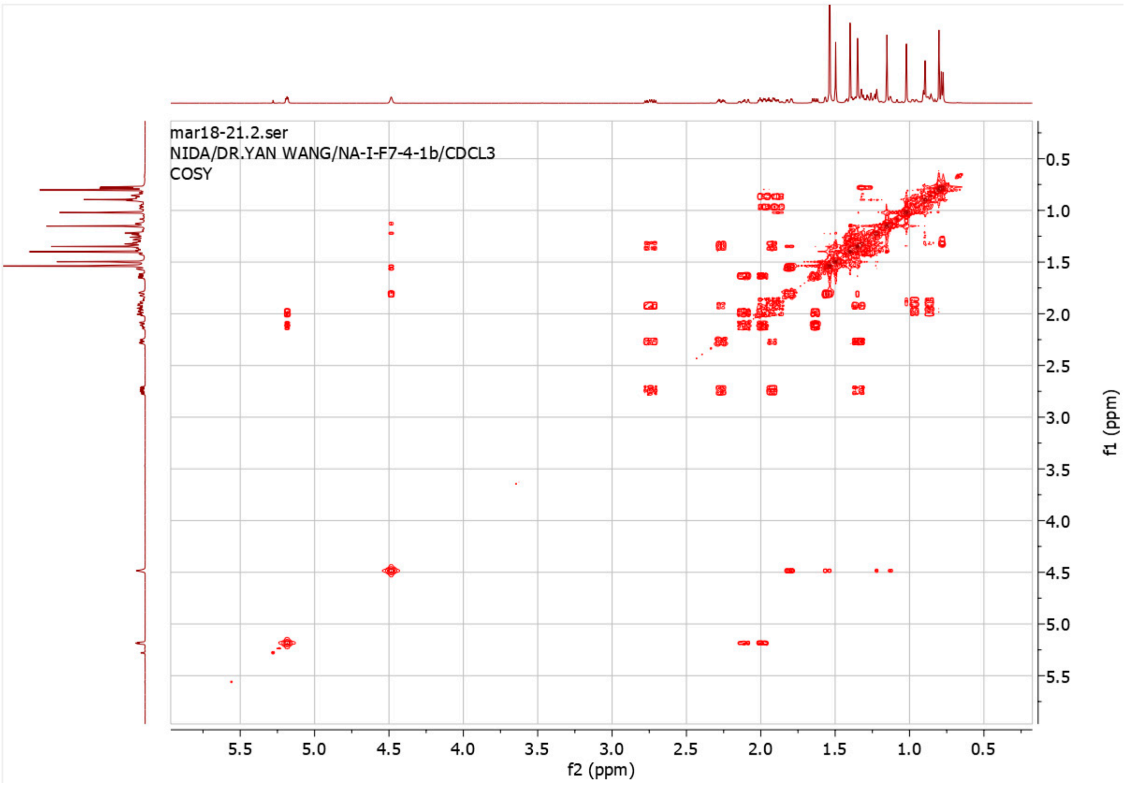

Figure S14. ^{1}H–^{1}H COSY spectrum-1 of compound **1**.

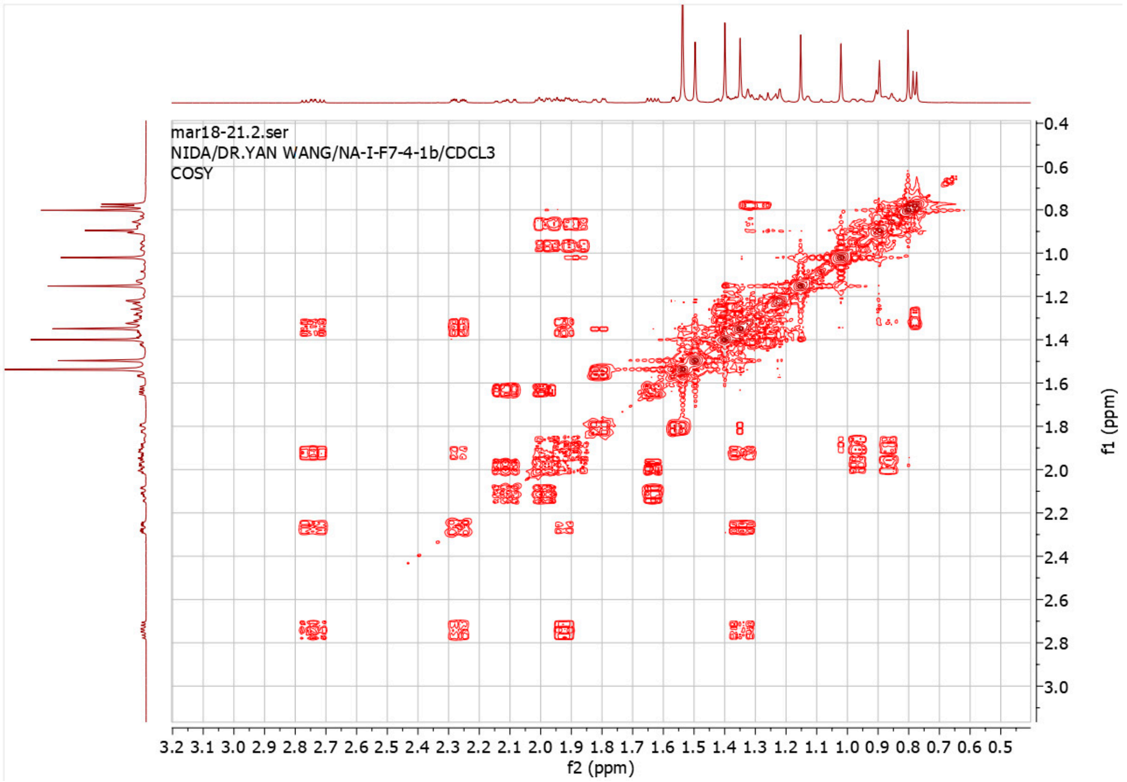

Figure S15. ^{1}H–^{1}H COSY spectrum-2 of compound **1**.

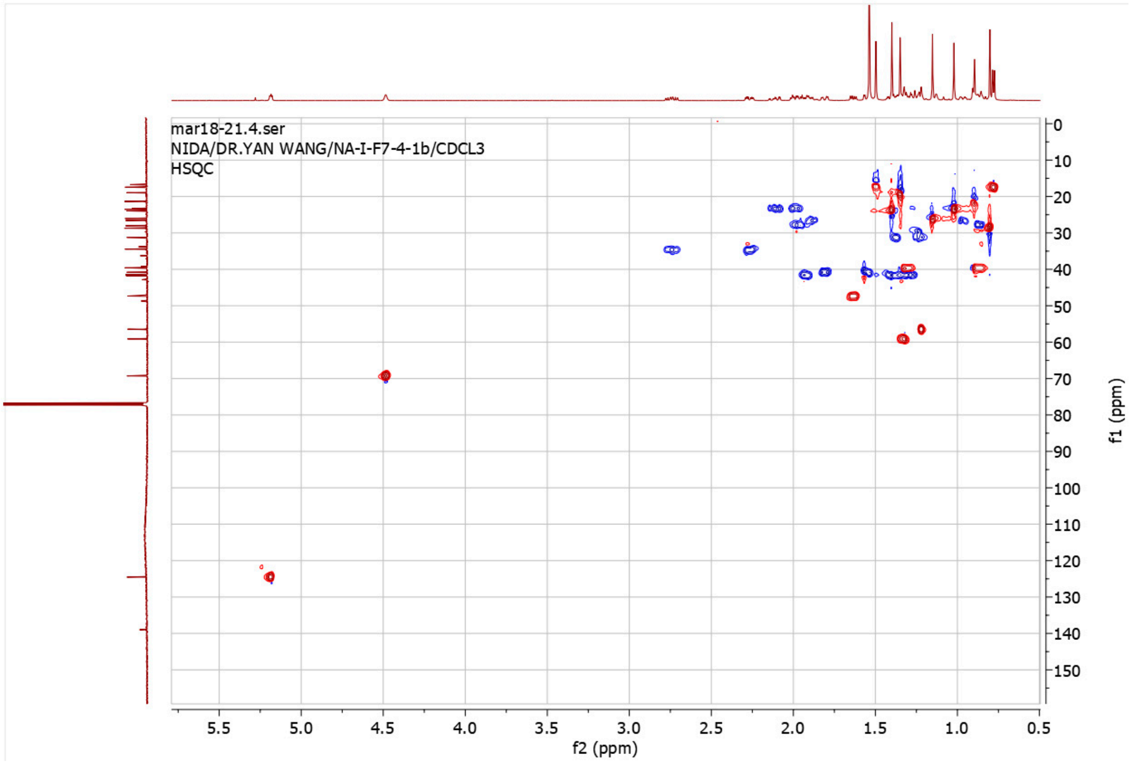

Figure S16. HSQC spectrum-1 of compound **1**.

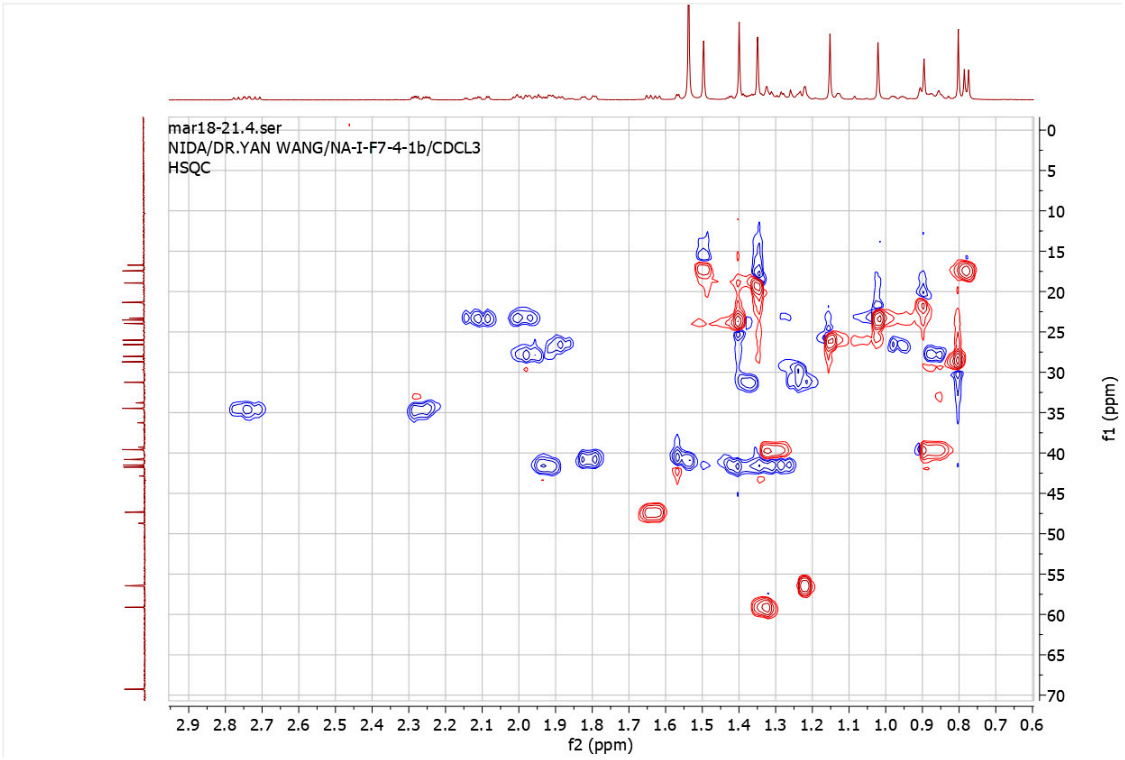

Figure S17. HSQC spectrum-2 of compound **1**.

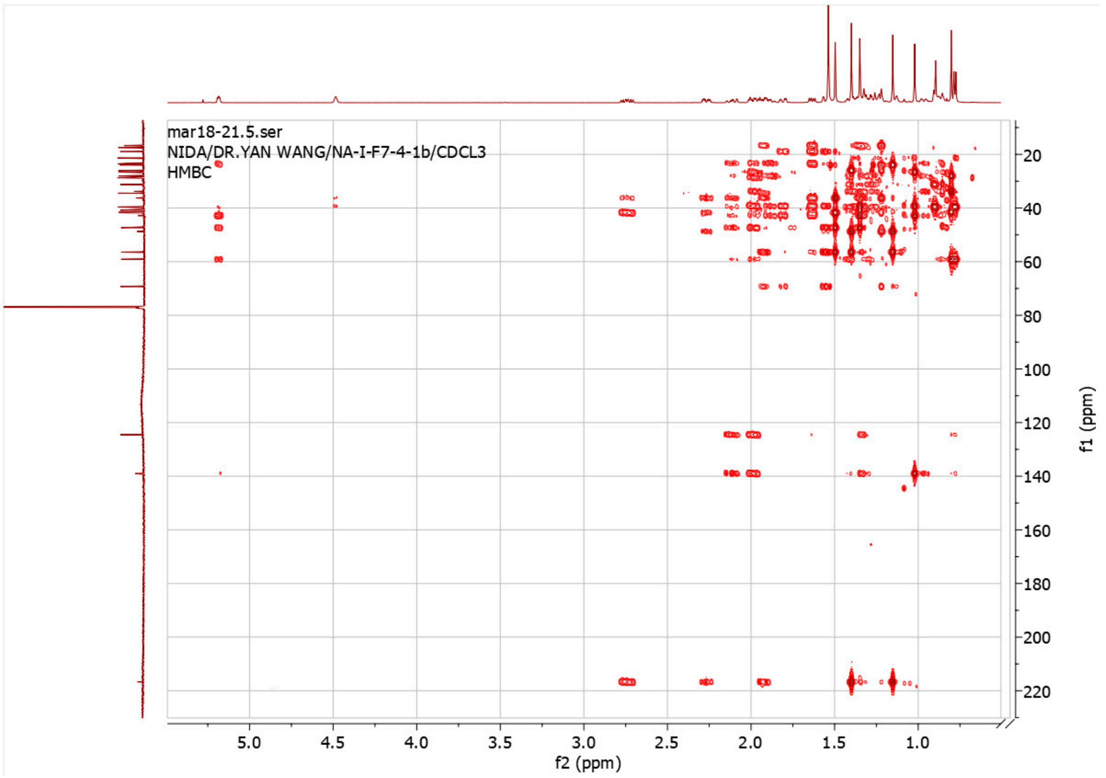

Figure S18. HMBC spectrum-1 of compound **1**.

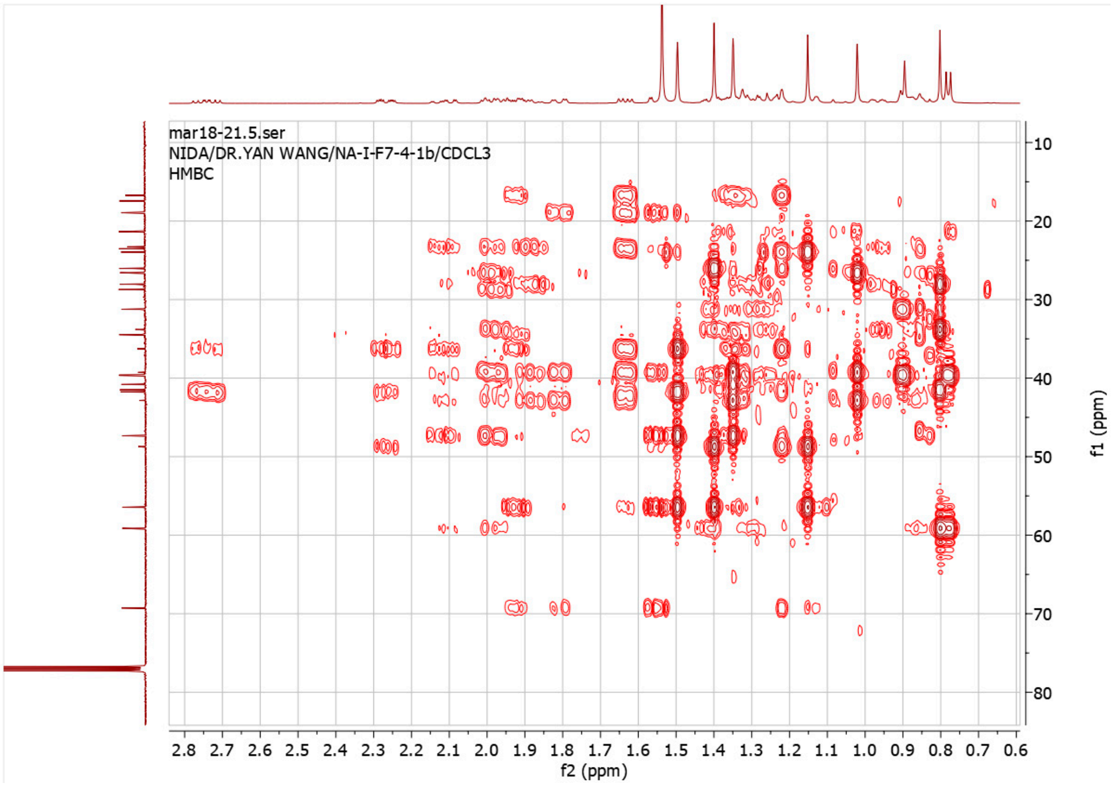

Figure S19. HMBC spectrum-2 of compound **1**.

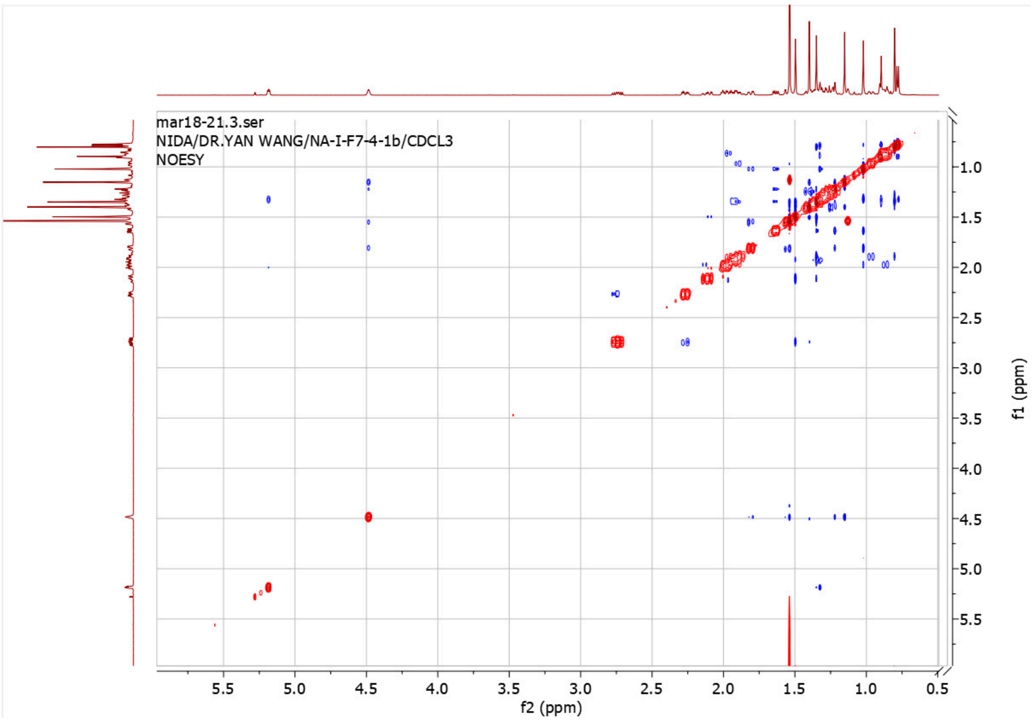

Figure S20. NOESY spectrum-1 of compound **1**.

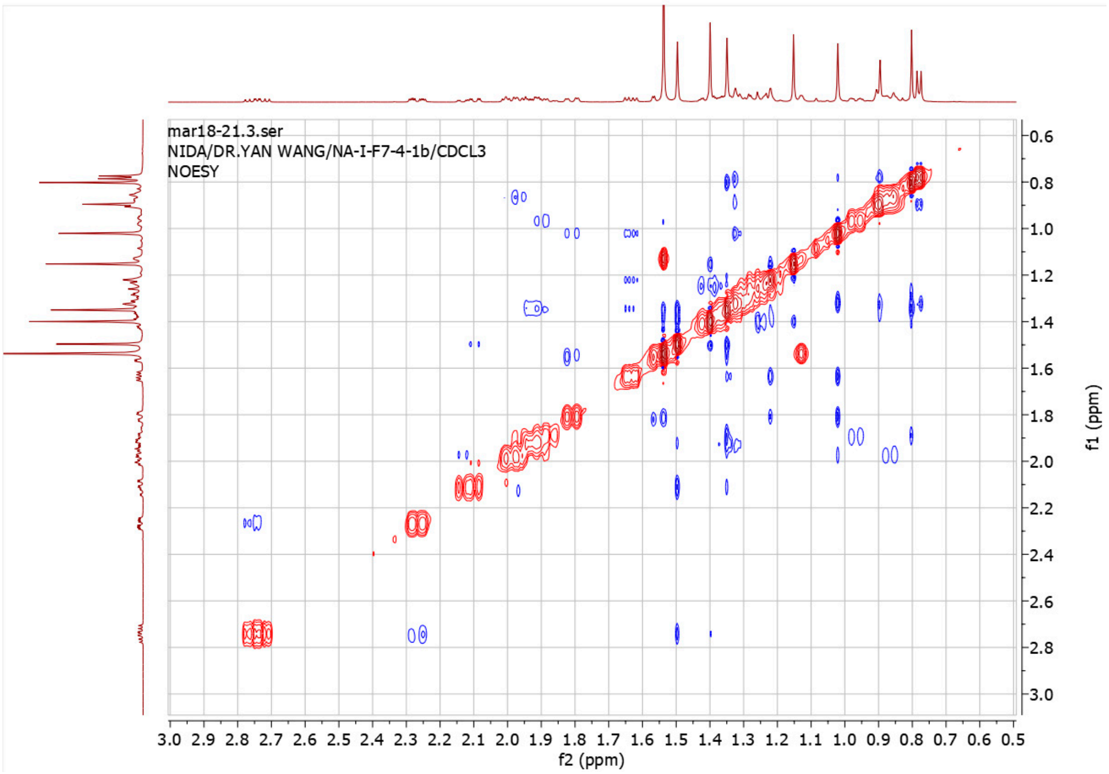

Figure S21. NOESY spectrum-2 of compound **1**.

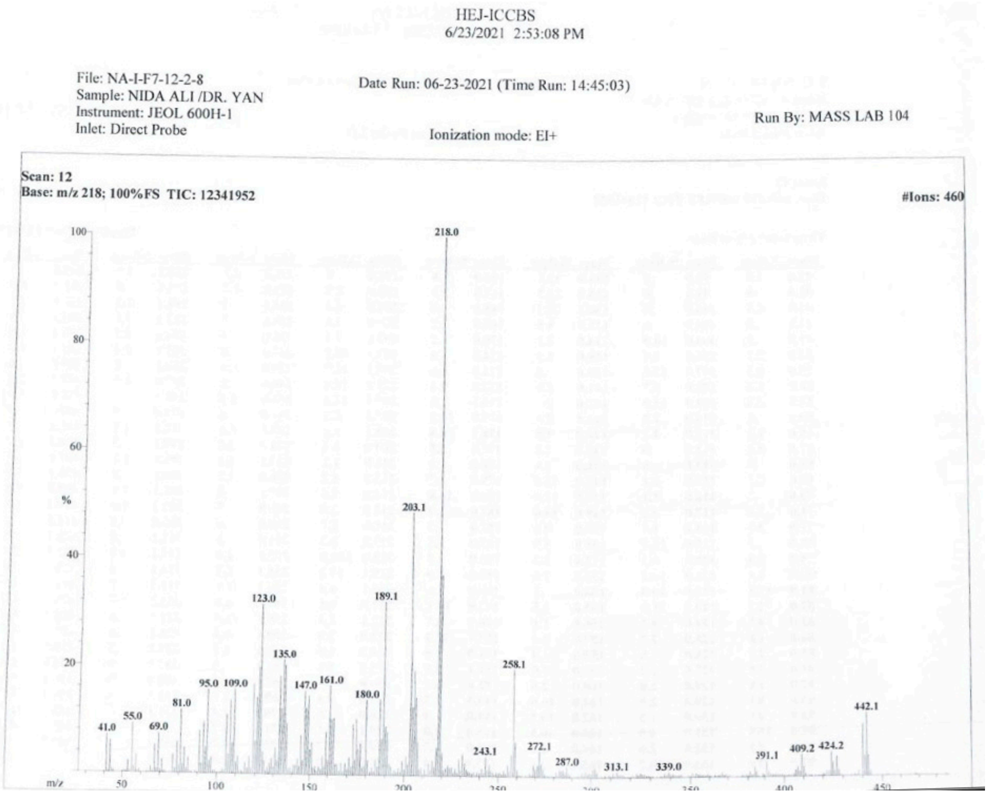

Figure S22. EI-MS spectrum of compound **2**.

Mass	Relative Intensity	Theoretical Mass	Delta [ppm]	Delta [mmu]	RDB	Composition
		213.1491	39.2	8.3	2.5	$C_{12}H_{21}O_3$
215.1713	1.4	215.1647	30.6	6.6	1.5	$C_{12}H_{23}O_3$
216.1825	1.2	216.1878	-24.6	-5.3	5.0	$C_{16}H_{24}$
217.1834	2.4	217.1804	13.8	3.0	0.5	$C_{12}H_{25}O_3$
218.2000	85.4	218.2035	-15.7	-3.4	4.0	$C_{16}H_{26}$
219.2033	17.1	219.2113	-36.2	-7.9	3.5	$C_{16}H_{27}$
220.2058	1.7					
225.1737	1.1					
229.1954	1.8	229.1956	-1.1	-0.3	5.5	$C_{17}H_{25}$
231.2082	1.4	231.2113	-13.3	-3.1	4.5	$C_{17}H_{27}$
232.1890	1.1	232.1827	26.9	6.2	5.0	$C_{16}H_{24}O_1$
243.2048	1.7	243.2113	-26.5	-6.4	5.5	$C_{18}H_{27}$
		243.1960	36.3	8.8	1.5	$C_{14}H_{27}O_3$
255.2125	1.3	255.2113	4.7	1.2	6.5	$C_{19}H_{27}$
257.2234	1.7	257.2269	-13.6	-3.5	5.5	$C_{19}H_{29}$
258.2372	8.4	258.2348	9.4	2.4	5.0	$C_{19}H_{30}$
259.2408	2.8	259.2426	-6.9	-1.8	4.5	$C_{19}H_{31}$
269.2204	1.2	269.2269	-24.4	-6.6	6.5	$C_{20}H_{29}$
		269.2117	32.3	8.7	2.5	$C_{16}H_{29}O_3$
270.2302	1.3	270.2348	-16.7	-4.5	6.0	$C_{20}H_{30}$
		270.2195	39.8	10.7	2.0	$C_{16}H_{30}O_3$
271.2322	1.1	271.2273	18.1	4.9	1.5	$C_{16}H_{31}O_3$
		271.2426	-38.1	-10.3	5.5	$C_{20}H_{31}$
272.2520	1.8	272.2504	5.7	1.6	5.0	$C_{20}H_{32}$
391.3352	1.8	391.3365	-3.1	-1.2	8.5	$C_{29}H_{43}$
		391.3212	35.8	14.0	4.5	$C_{25}H_{43}O_3$
406.3618	1.8	406.3600	4.5	1.8	8.0	$C_{30}H_{46}$
407.3505	1.1	407.3525	-5.0	-2.0	3.5	$C_{26}H_{47}O_3$
422.3493	1.3	422.3549	-13.2	-5.6	8.0	$C_{30}H_{46}O_1$
		422.3396	23.0	9.7	4.0	$C_{26}H_{46}O_4$
424.3649	2.3	424.3705	-13.2	-5.6	7.0	$C_{30}H_{48}O_1$
		424.3553	22.7	9.7	3.0	$C_{26}H_{48}O_4$
440.3688	1.4	440.3654	7.7	3.4	7.0	$C_{30}H_{48}O_2$
442.3832	2.3	442.3811	4.9	2.2	6.0	$C_{30}H_{50}O_2$
		442.3658	39.4	17.4	2.0	$C_{26}H_{50}O_5$

m/z

(calc. 442.3811)

Figure S23. HR-EI-MS spectrum of compound **2**.

THERMO ELECTRON ~ VISIONpro SOFTWARE V4.10

Operator Name	Zainab Rizvi	Date of Report	8/31/2021
Department	Analytical Lab,. Nanotechnology Cntr.	Time of Report	2:49:20PM
Organization	ICCBS ,University of Kar.PAKISTAN		
Information	Aamir/Dr.Yan		

Scan Graph

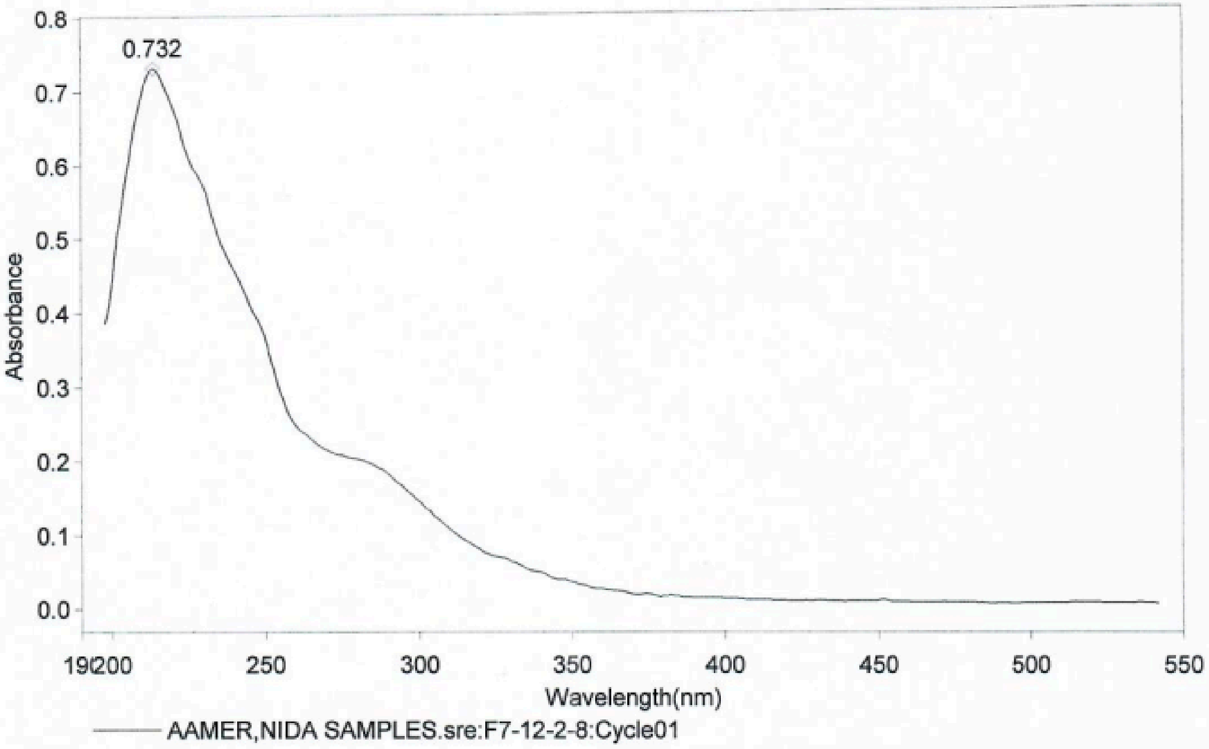

Results Table - AAMER,NIDA SAMPLES.sre,F7-12-2-8,Cycle01

nm	A	**Peak Pick Method**
214.00	0.732	Find 8 Peaks Above -3.0000 A
		Start Wavelength 190.00 nm
		Stop Wavelength 300.00 nm
		Sort By Wavelength
Sensitivity	Very High	

Figure S24. UV spectrum of compound **2**.

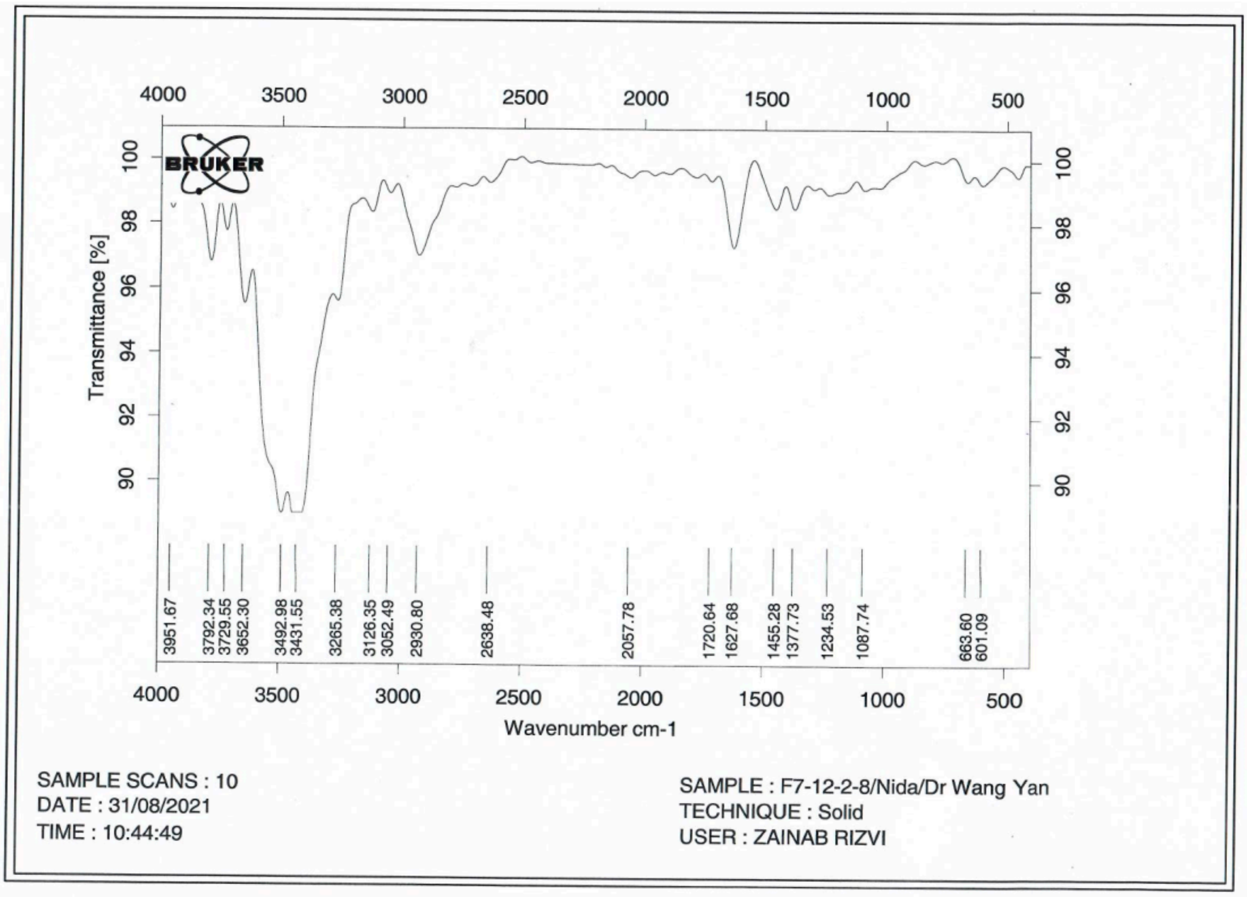

Figure S25. IR spectrum of compound **2**.

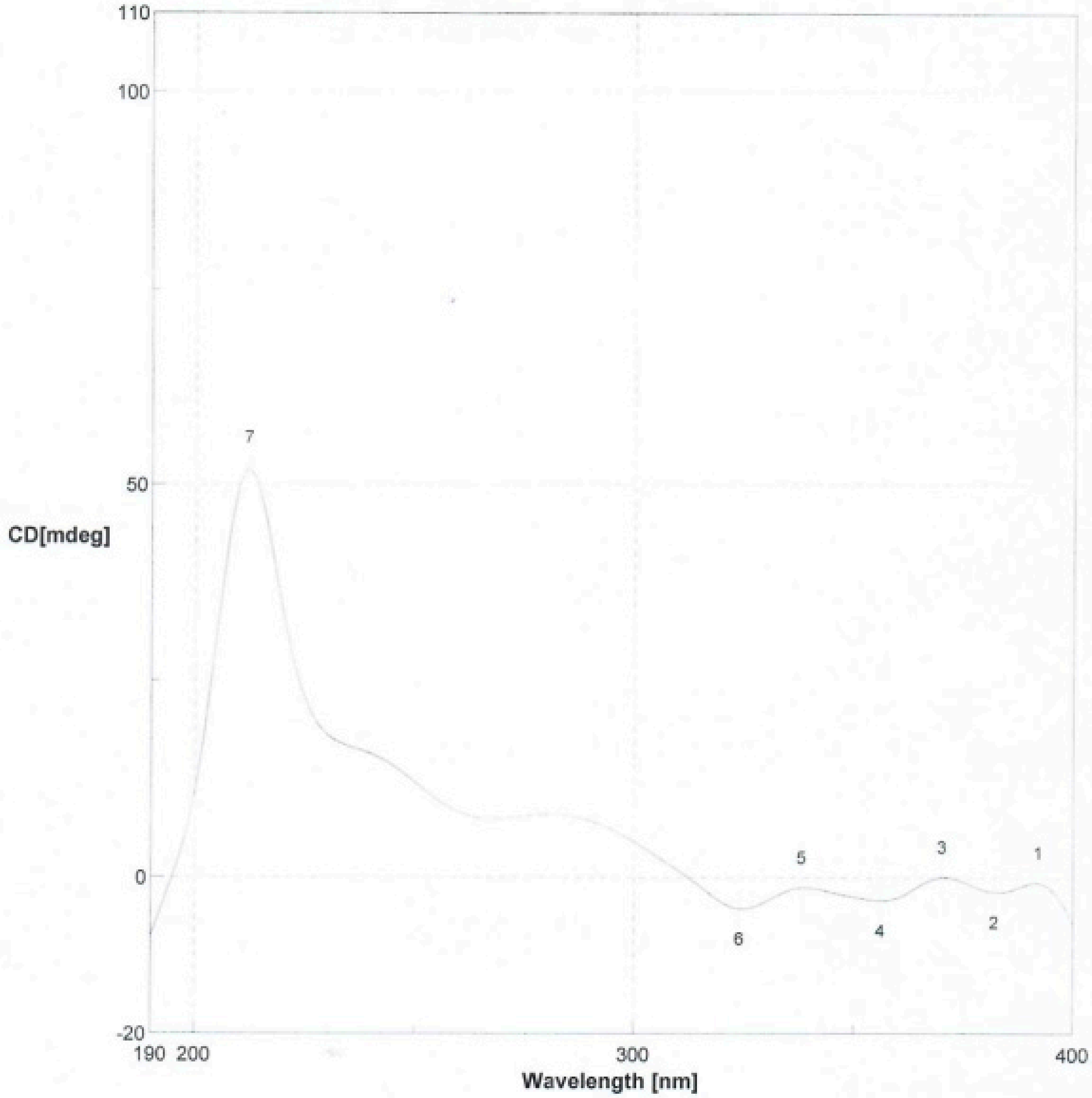

Date/Time	8/30/2021 10:54AM
Operator	ZAINAB RIZVI
File Name	Memory#3
Sample Name	F7-12-2-8
Comment	Nida/Dr.Yan

No.	nm	CD[mdeg]	No.	nm	CD[mdeg]	No.	nm	CD[mdeg]
1	392	-0.645019	2	382	-1.89053	3	370	0.079522
4	356	-2.94504	5	338	-1.28881	6	324	-4.00665
7	212	52.1492						

Figure S26. CD spectrum of compound **2**.

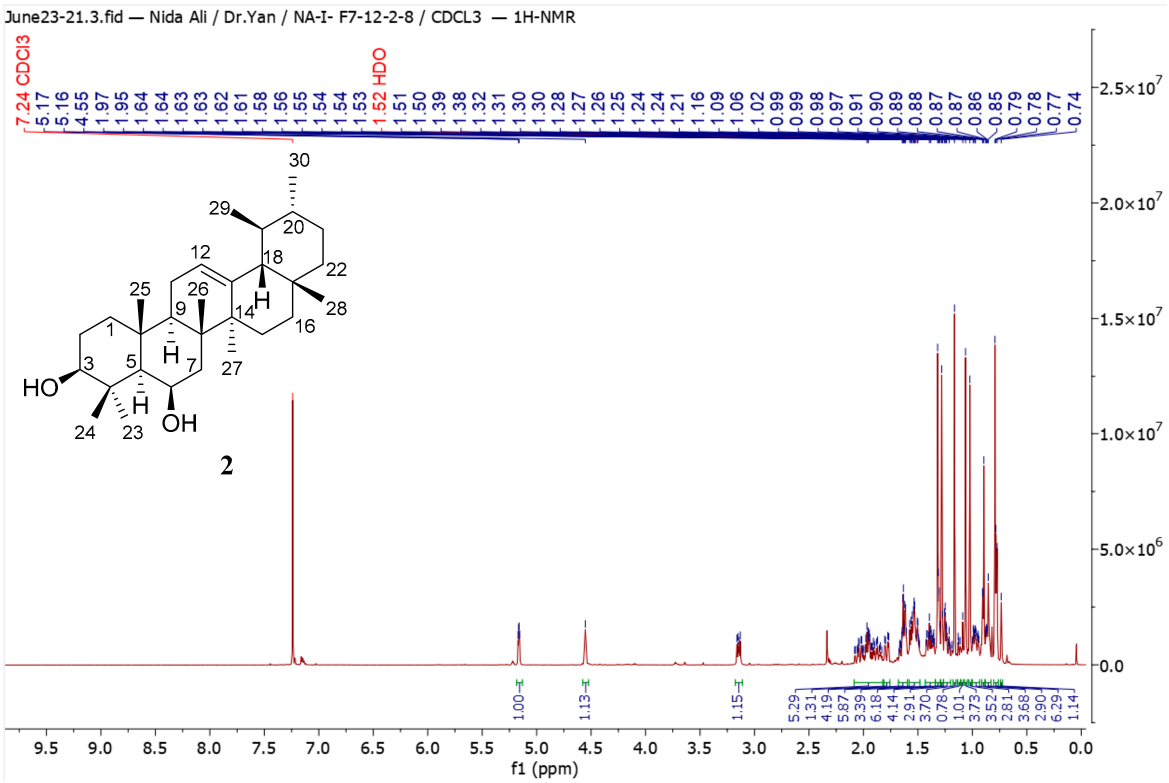

Figure S27. ^{1}H NMR spectrum of compound **2** (500 MHz, $CDCl_3$).

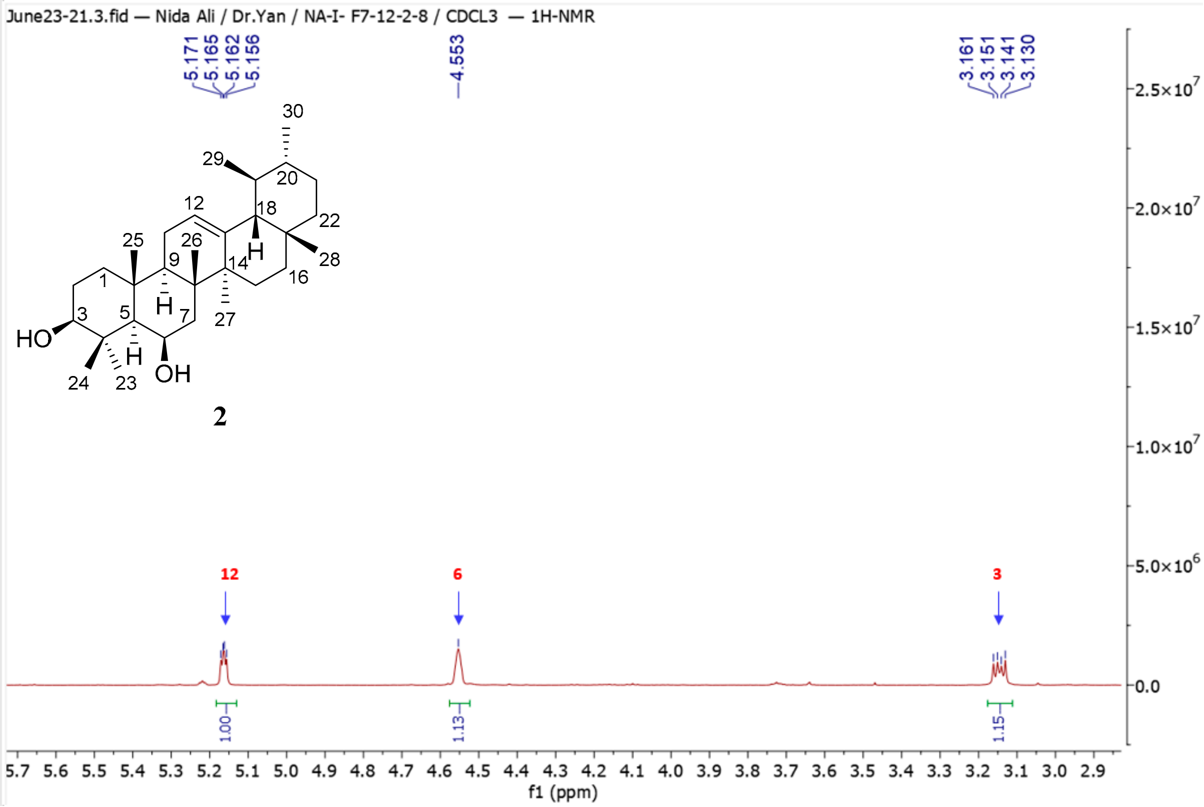

Figure S28. ^{1}H NMR assignment-1 of compound **2**.

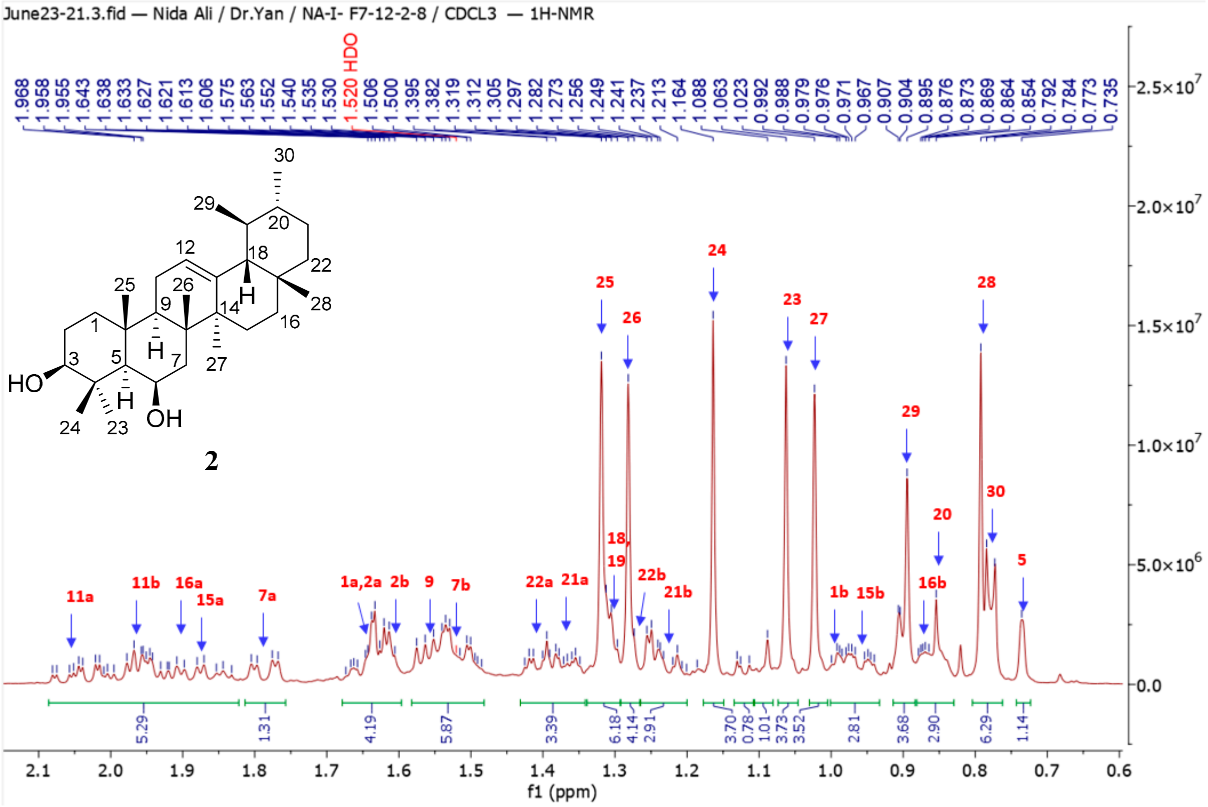

Figure S29. ^{1}H NMR assignment-2 of compound **2**.

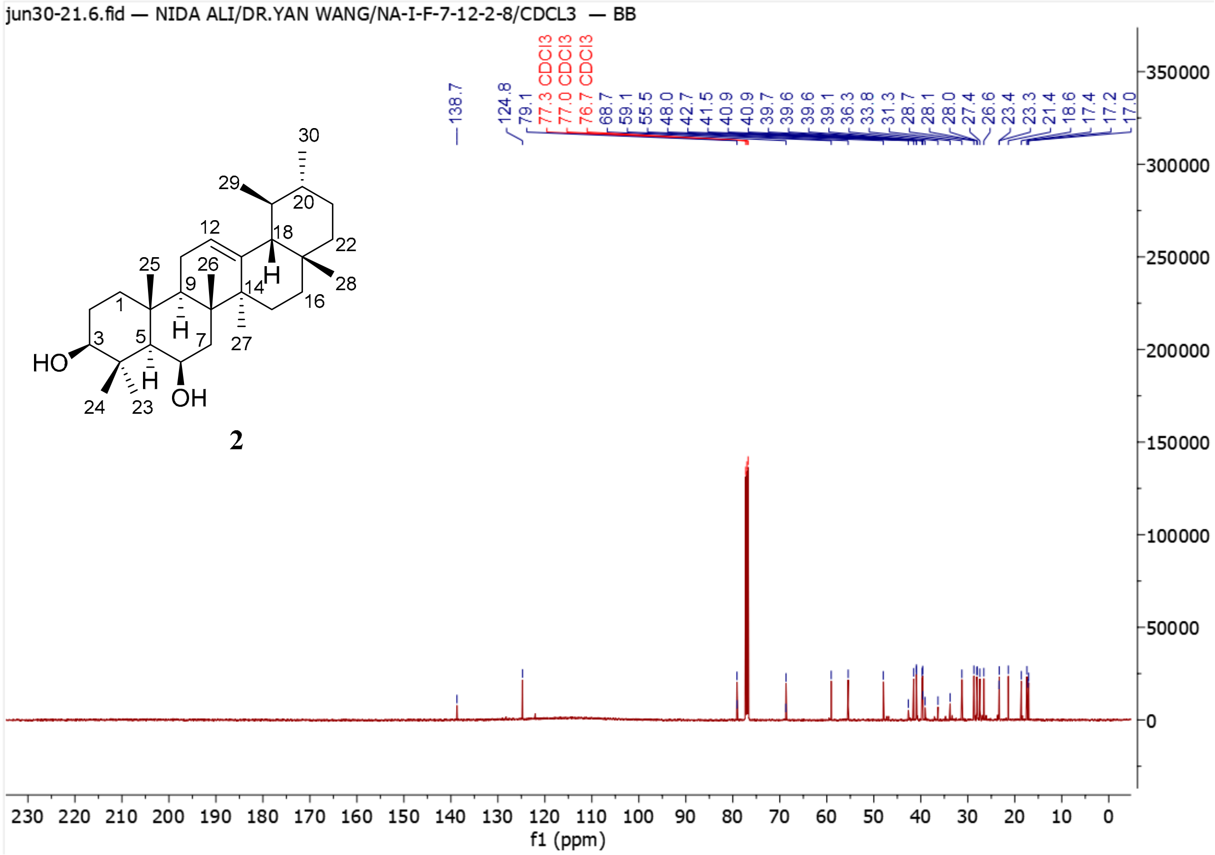

Figure S30. ^{13}C NMR spectrum of compound **2** (150 MHz, $CDCl_3$).

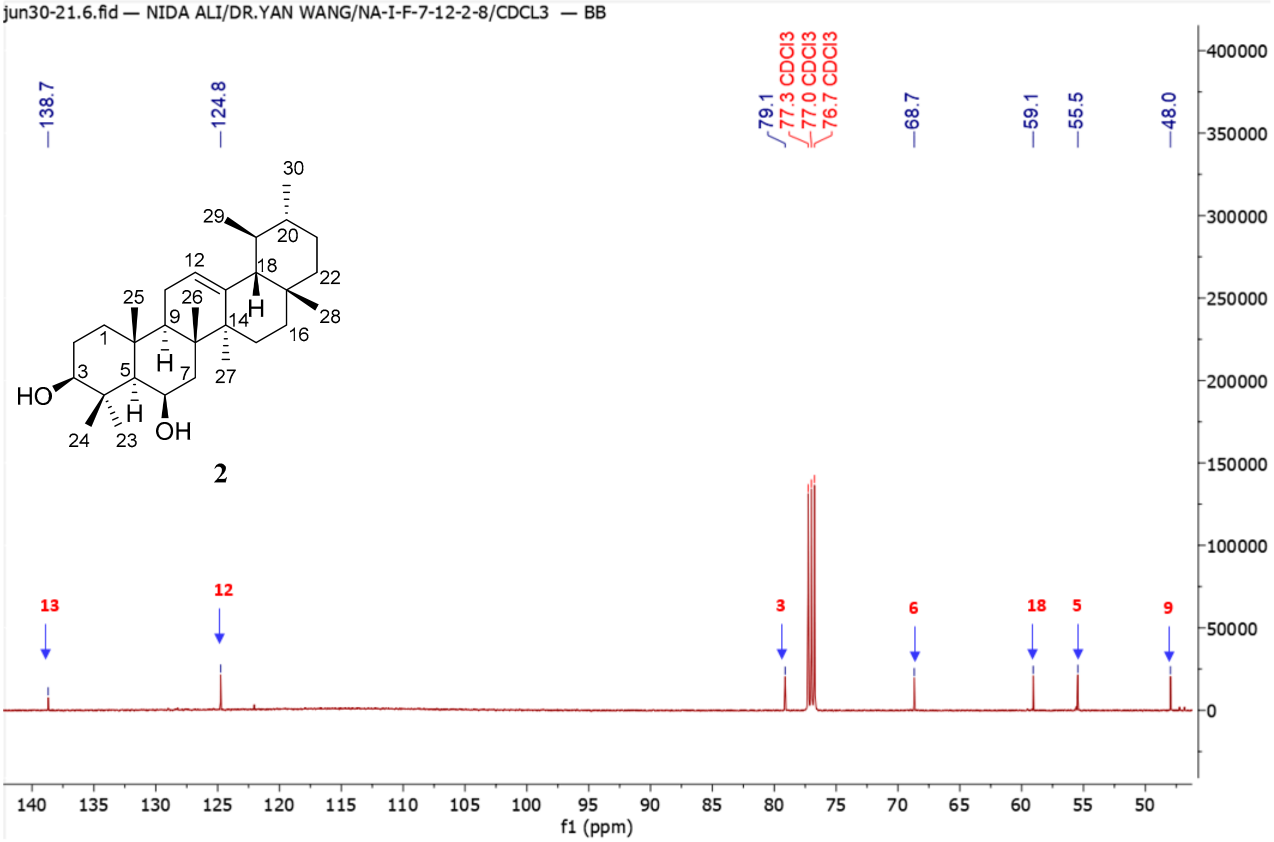

Figure S31. ^{13}C NMR assignment-1 of compound **2**.

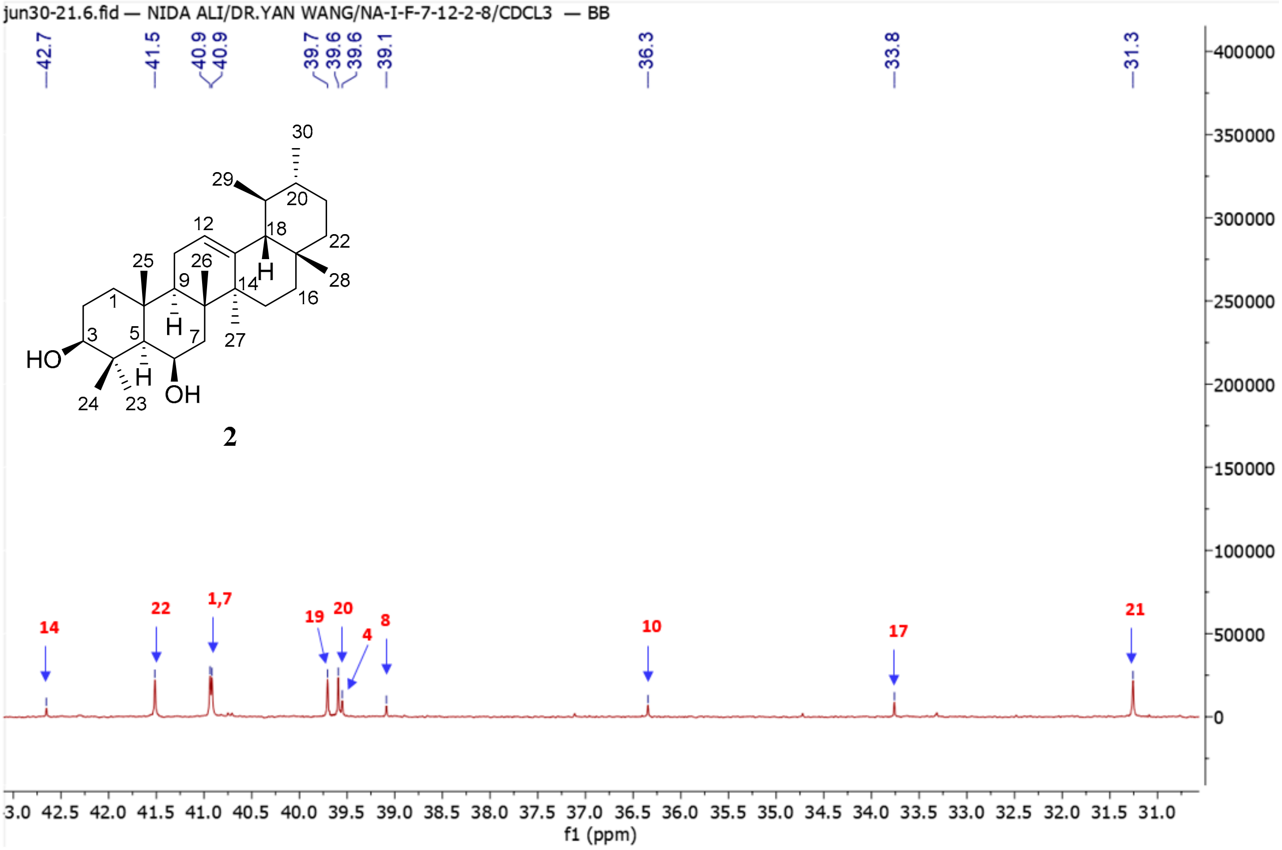

Figure S32. ^{13}C NMR assignment-2 of compound **2**.

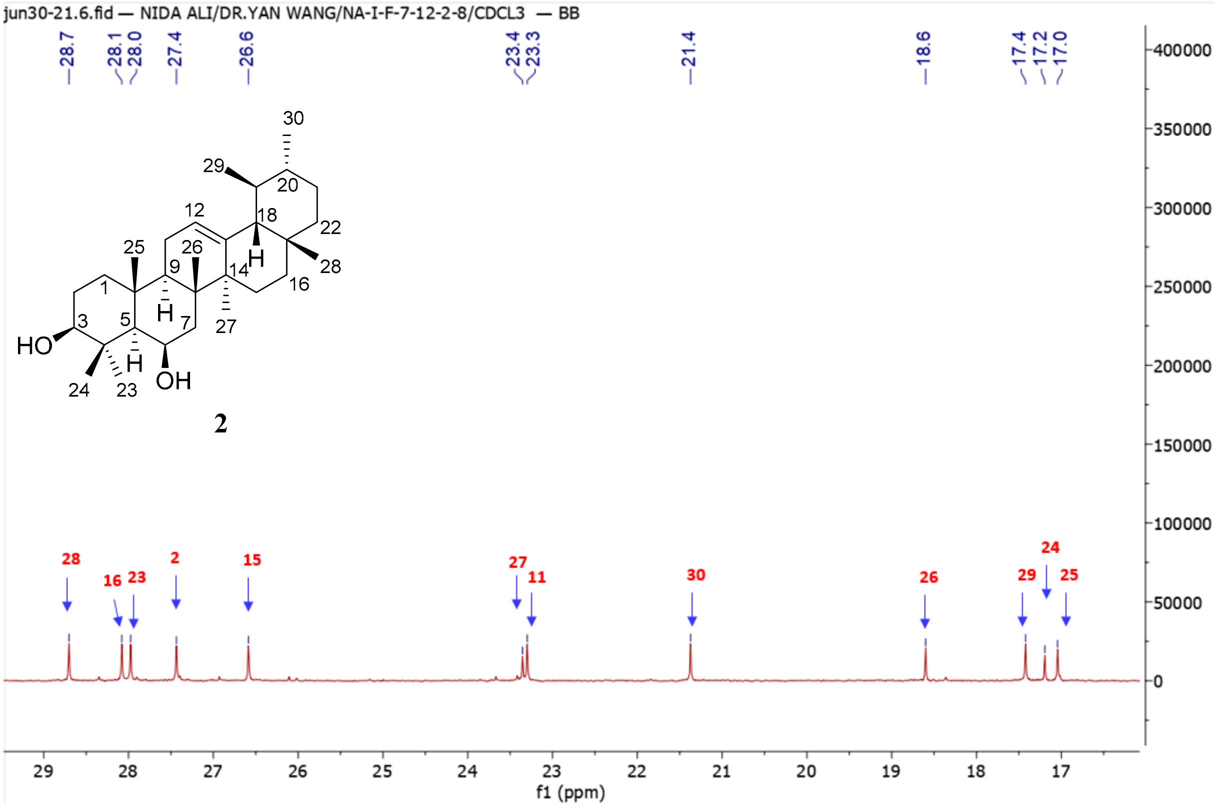

Figure S33. ^{13}C NMR assignment-3 of compound **2**.

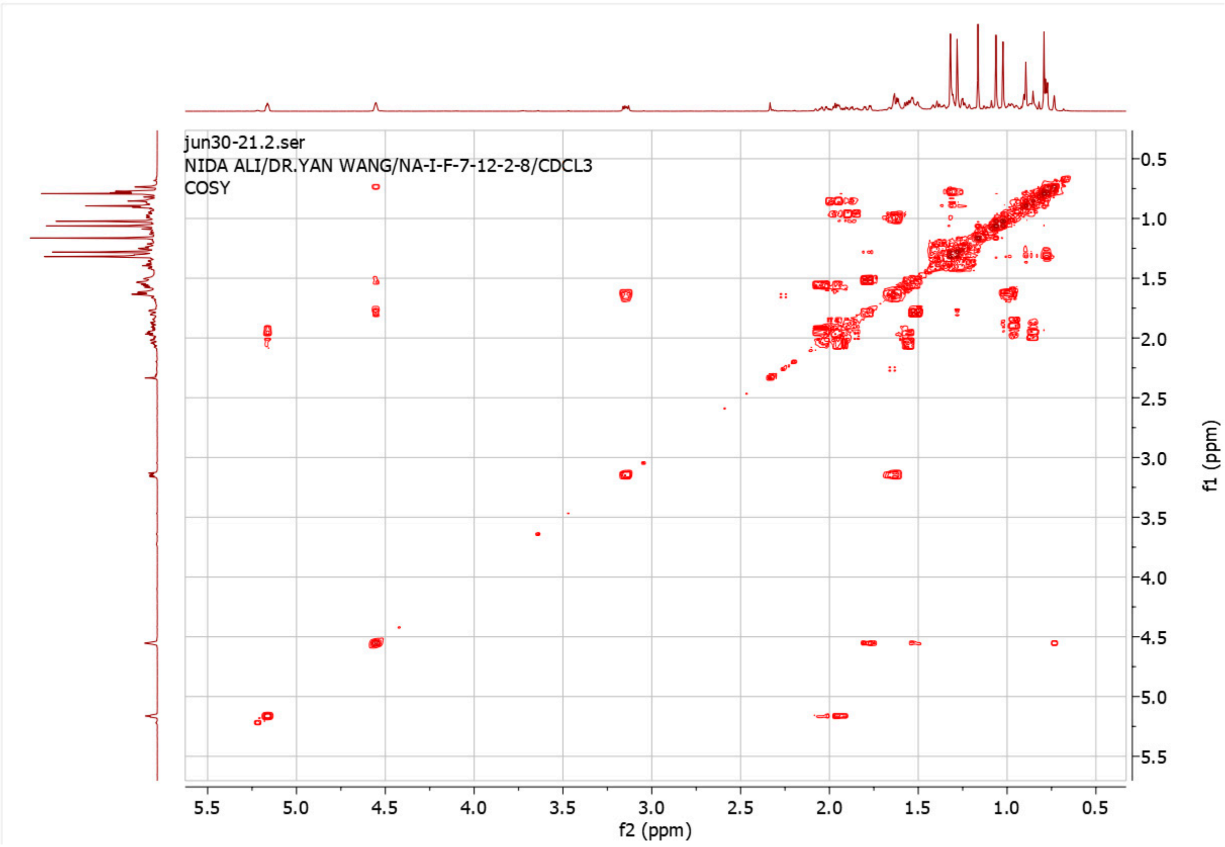

Figure S34. ^{1}H–^{1}H COSY spectrum-1 of compound **2**.

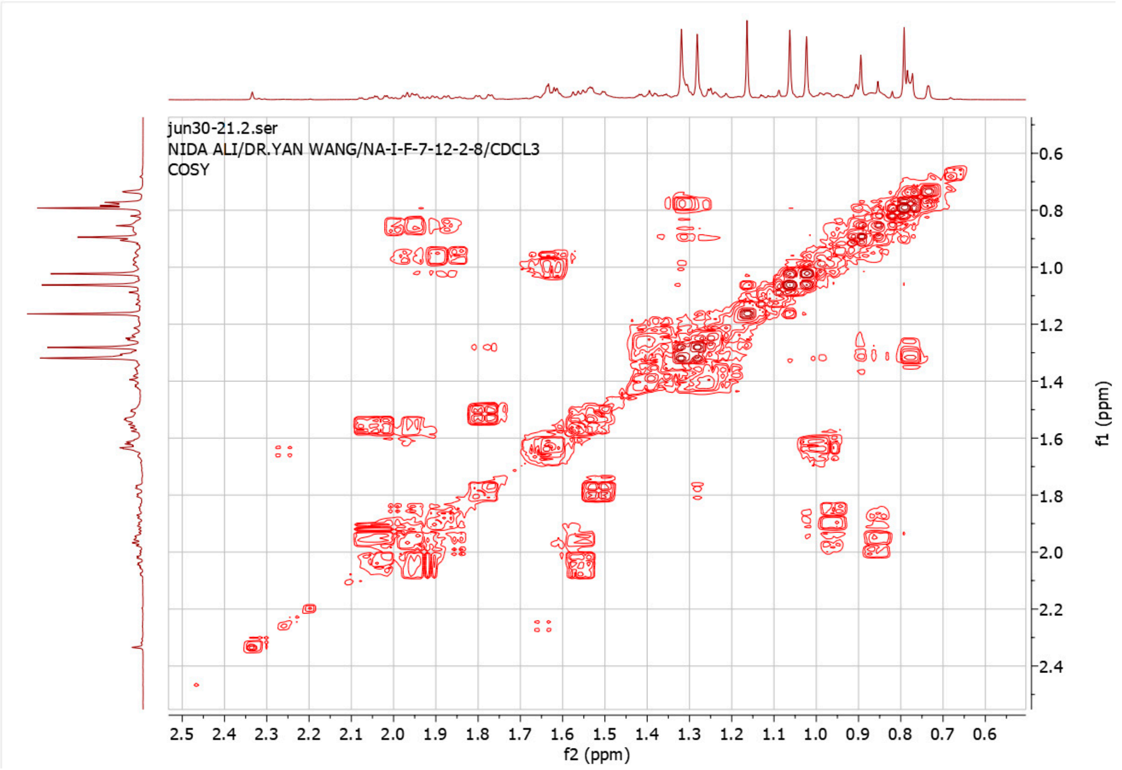

Figure S35. ^{1}H–^{1}H COSY spectrum-2 of compound **2**.

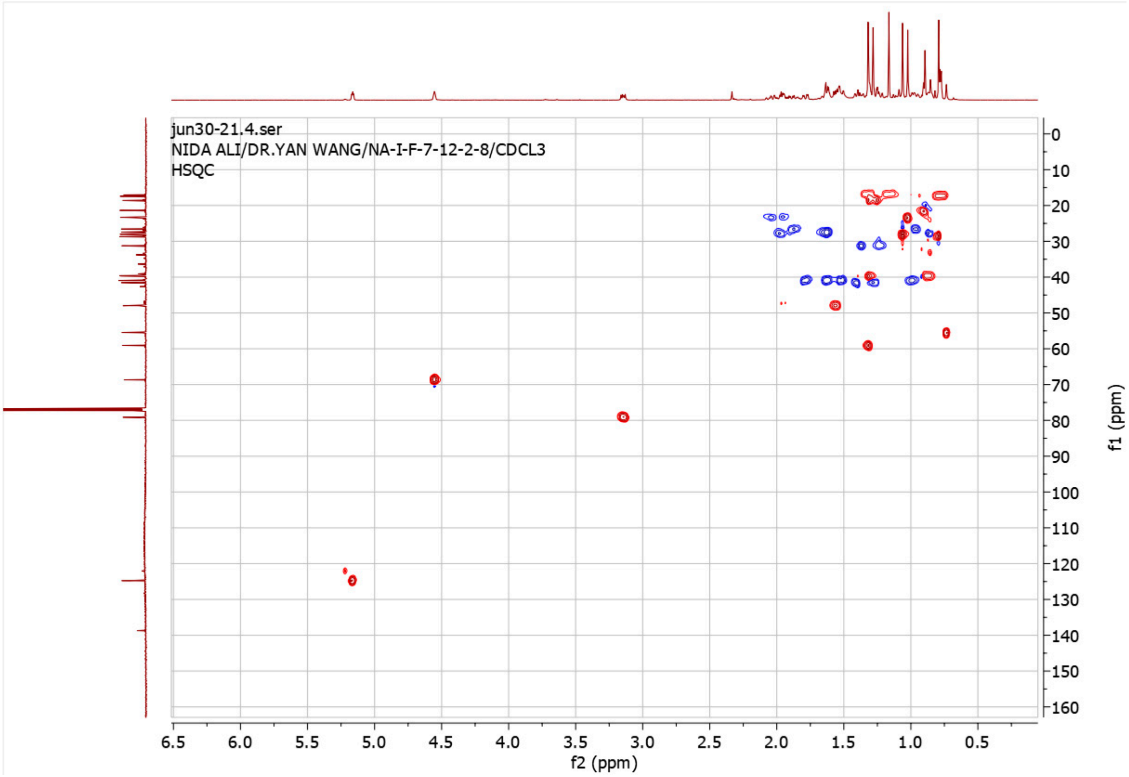

Figure S36. HSQC spectrum-1 of compound **2**.

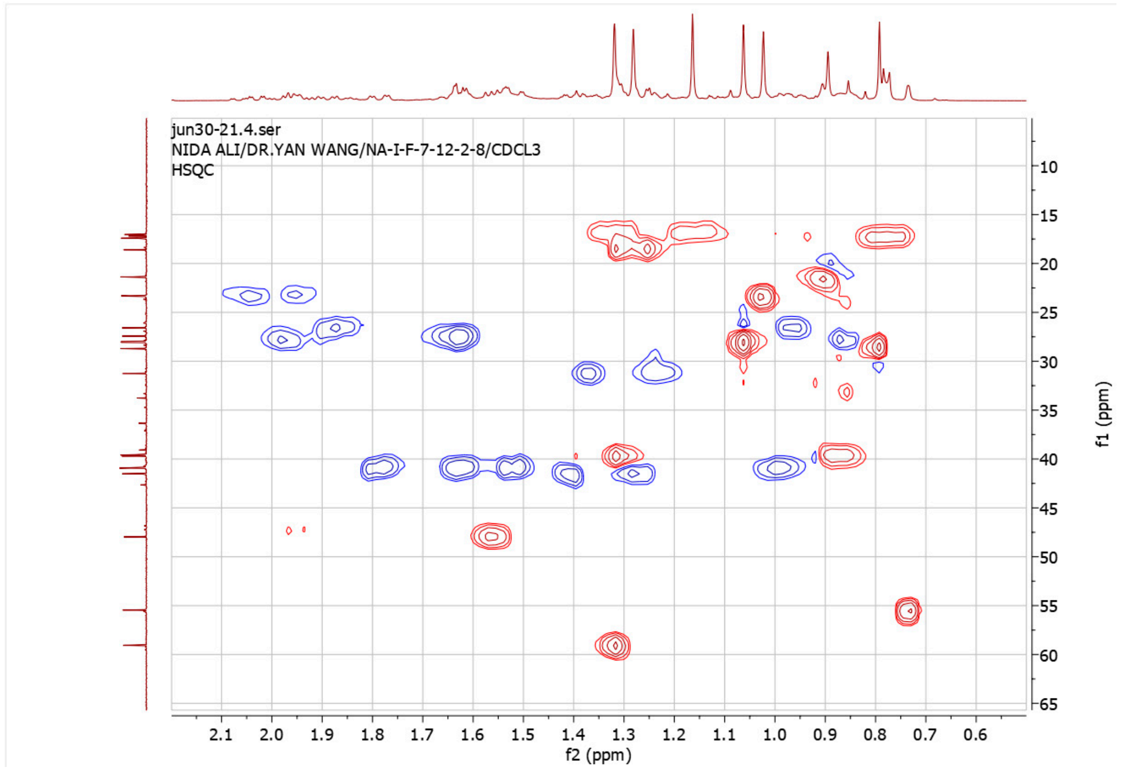

Figure S37. HSQC spectrum-2 of compound **2**.

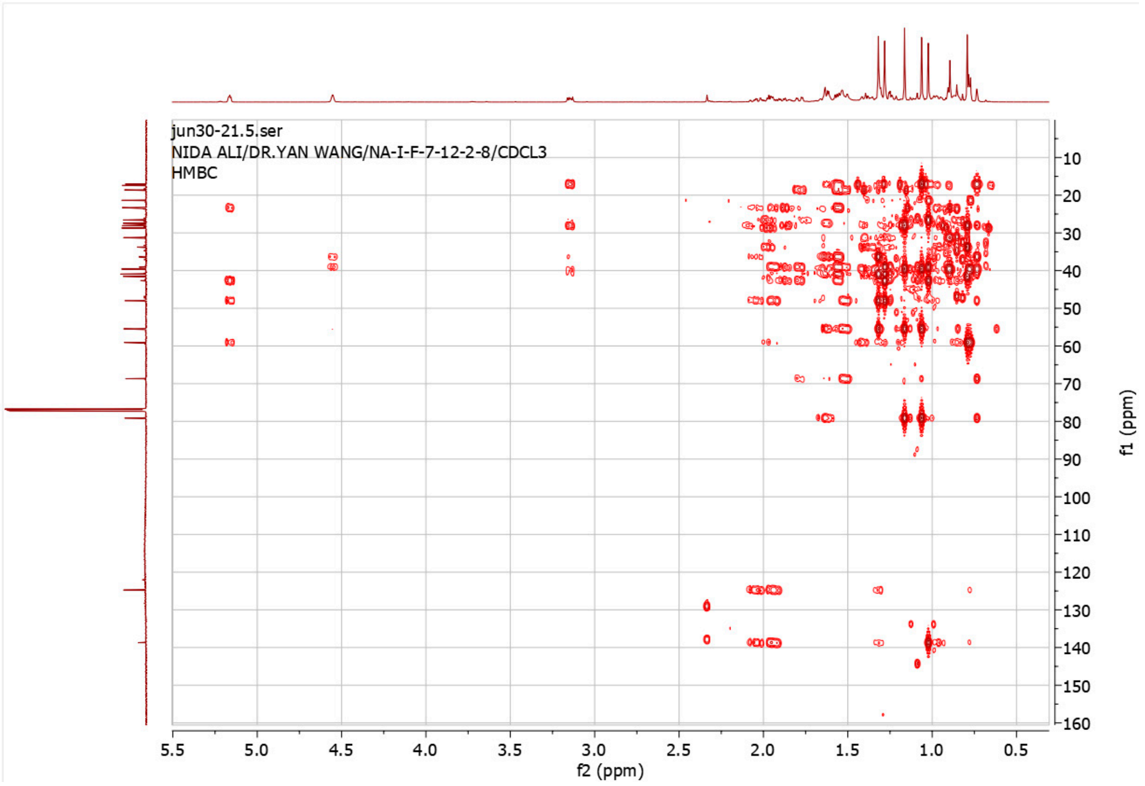

Figure S38. HMBC spectrum-1 of compound **2**.

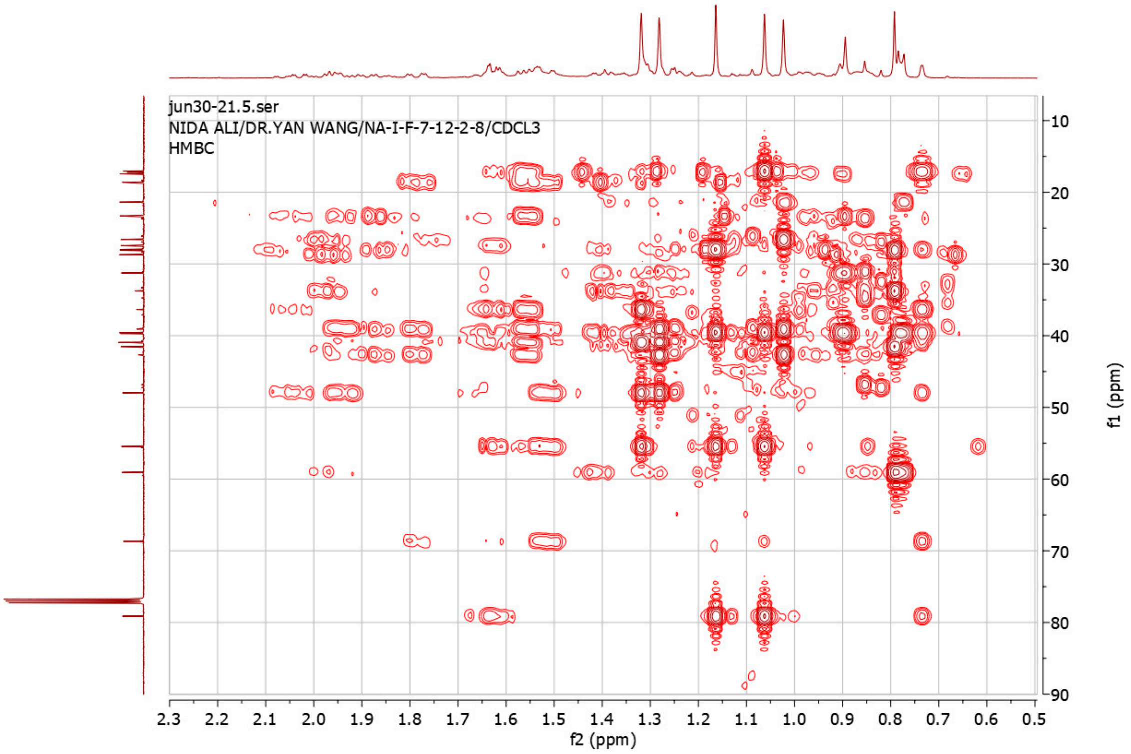

Figure S39. HMBC spectrum-2 of compound **2**.

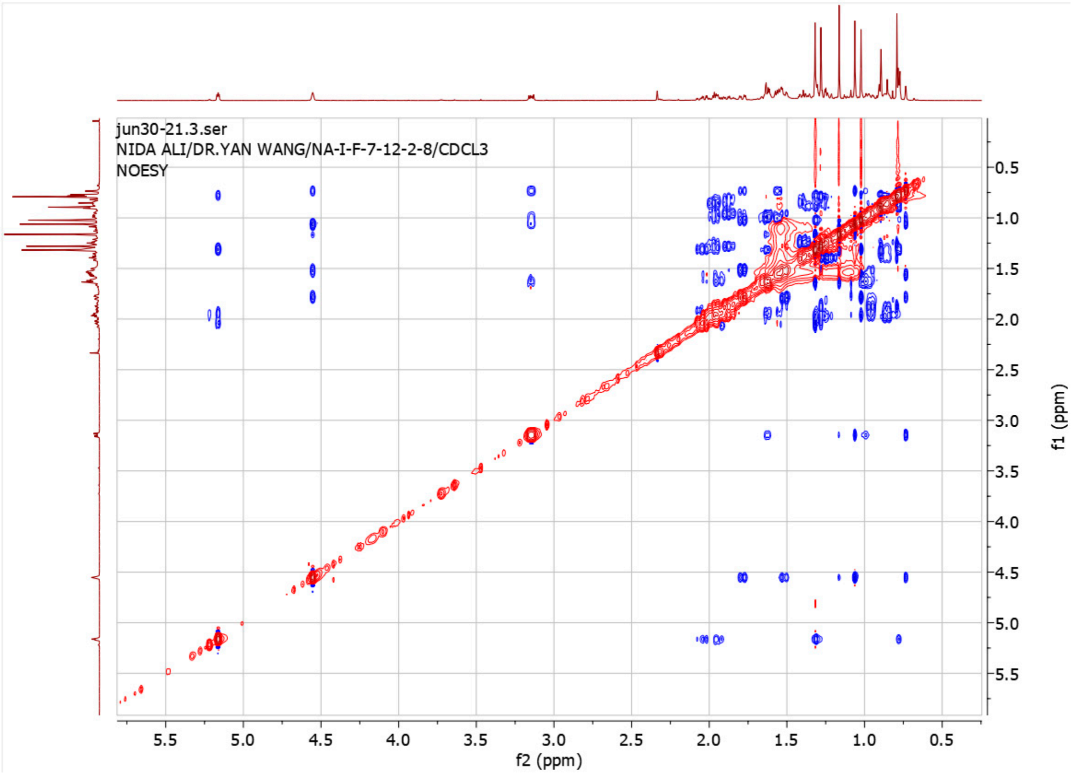

Figure S40. NOESY spectrum-1 of compound **2**.

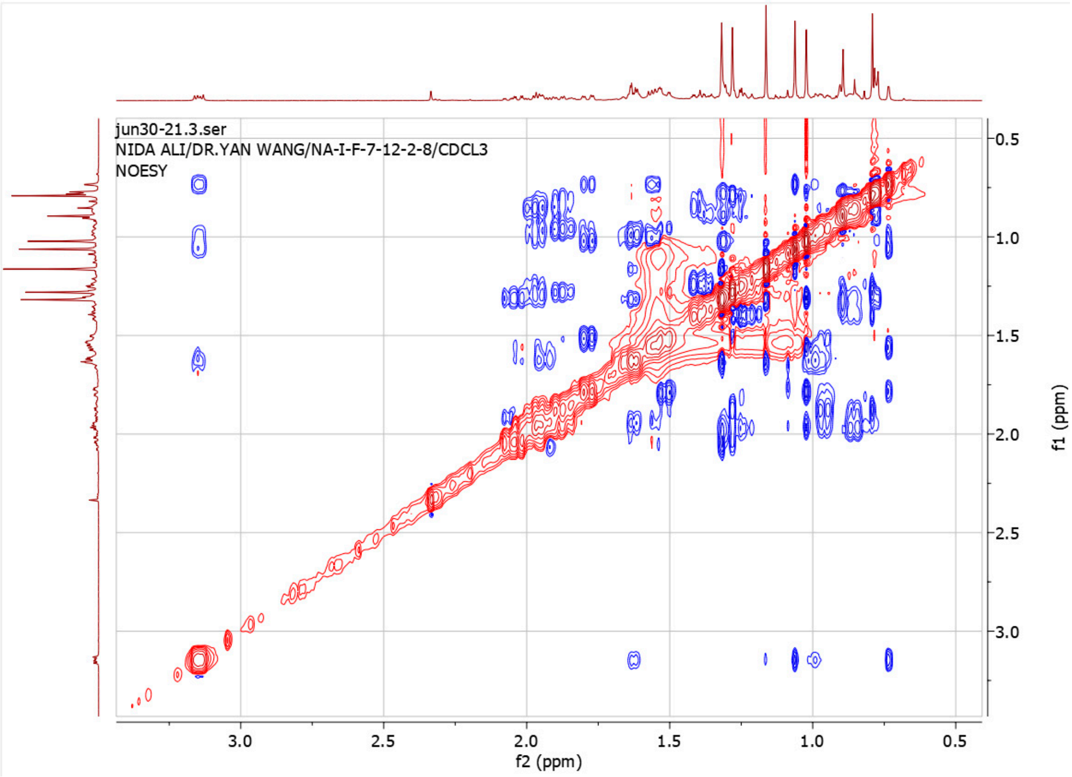

Figure S41. NOESY spectrum-2 of compound **2**.
